# Investigation of Earth-Abundant Metal Salts for the Inhibition of Asphalt-Derived Volatile Organic Compounds

**DOI:** 10.1021/acsomega.4c02095

**Published:** 2024-05-13

**Authors:** Harpreet Kaur, Reem Nsouli, Gabriella Cerna, Saba Shariati, Marco Flores, Elham H. Fini, Laura K. G. Ackerman-Biegasiewicz

**Affiliations:** † Department of Chemistry, Emory University, Atlanta, Georgia 30322, United States; ‡ School of Molecular Sciences, Arizona State University, 660 S. College Avenue, Tempe, Arizona 85287-3005, United States; § School of Sustainable Engineering and the Built Environment, Arizona State University, 660 S. College Avenue, Tempe, Arizona 85287-3005, United States

## Abstract

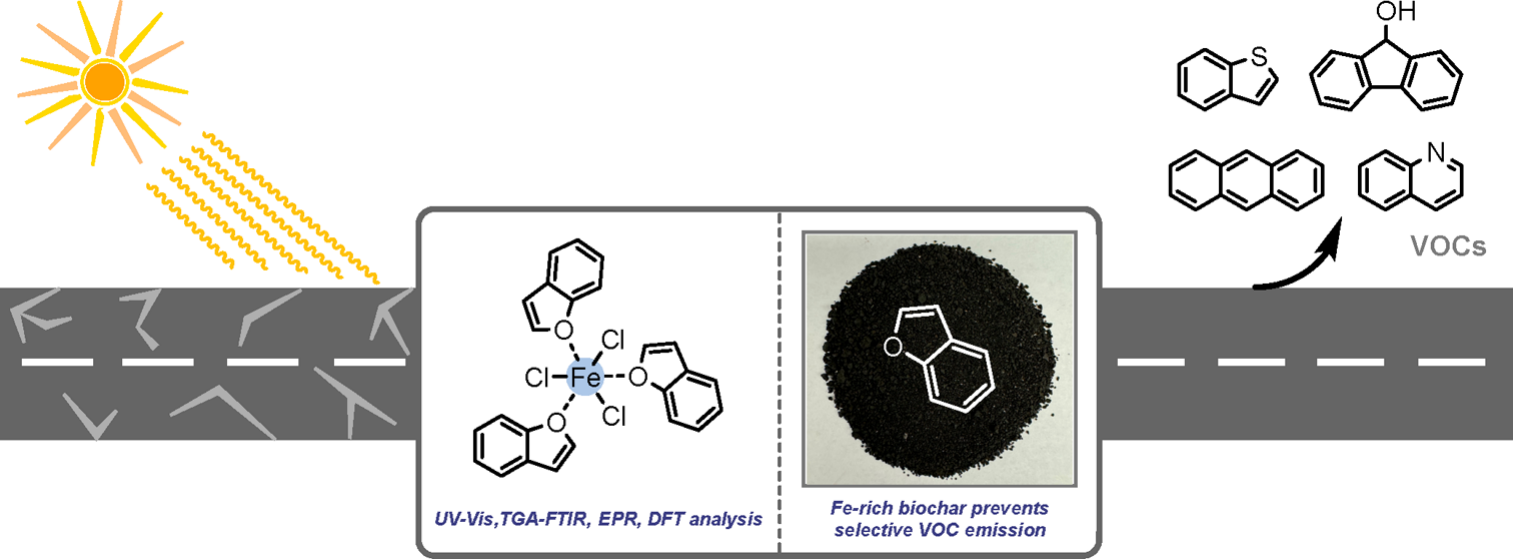

Asphalt is used globally in construction for roads, pavements, and buildings; however, as a fossil-derived material, it is known to generate volatile organic compounds (VOCs) upon exposure to heat and light that can be harmful to human health. Several heterogeneous strategies have been reported for the inhibition of these VOCs; however, the direct use of inexpensive, accessible Earth-Abundant metals has not been extensively explored. In this study, simple metal salts are examined for their coordination capability toward asphalt-derived VOCs. From UV–visible (UV–vis) spectroscopic studies, FeCl_3_ emerged relative to other metal salts (metal = Mn, Co, Ni, Cu, Zn) as a promising candidate for the adsorption and retention of Lewis basic compounds. Coordination of an example oxygen-containing VOC, benzofuran (Bf), to Fe yielded a paramagnetic semi-octahedral complex Fe(Bf)_3_Cl_3_. Evaluation by thermal gravimetric analysis (TGA) coupled to infrared spectroscopy (IR) demonstrated that the complex was stable up to 360 °C. Spectroscopic evaluation demonstrated the stability of the complex upon visible light irradiation and in the presence of a variety of organic pollutants. The potential application of Fe was demonstrated by subjecting biochar to FeCl_3_ followed by the addition of Bf. It was discovered that this Fe-rich biochar was successful at adsorbing Bf suggesting the possibility of introducing Fe to biochar late-stage in processing to deter asphalt degradation and VOC emissions. An understanding of the binding and stability of Fe salts to VOCs provides insight into how a sustainable infrastructure can be achieved.

## Introduction

**Figure 1:**
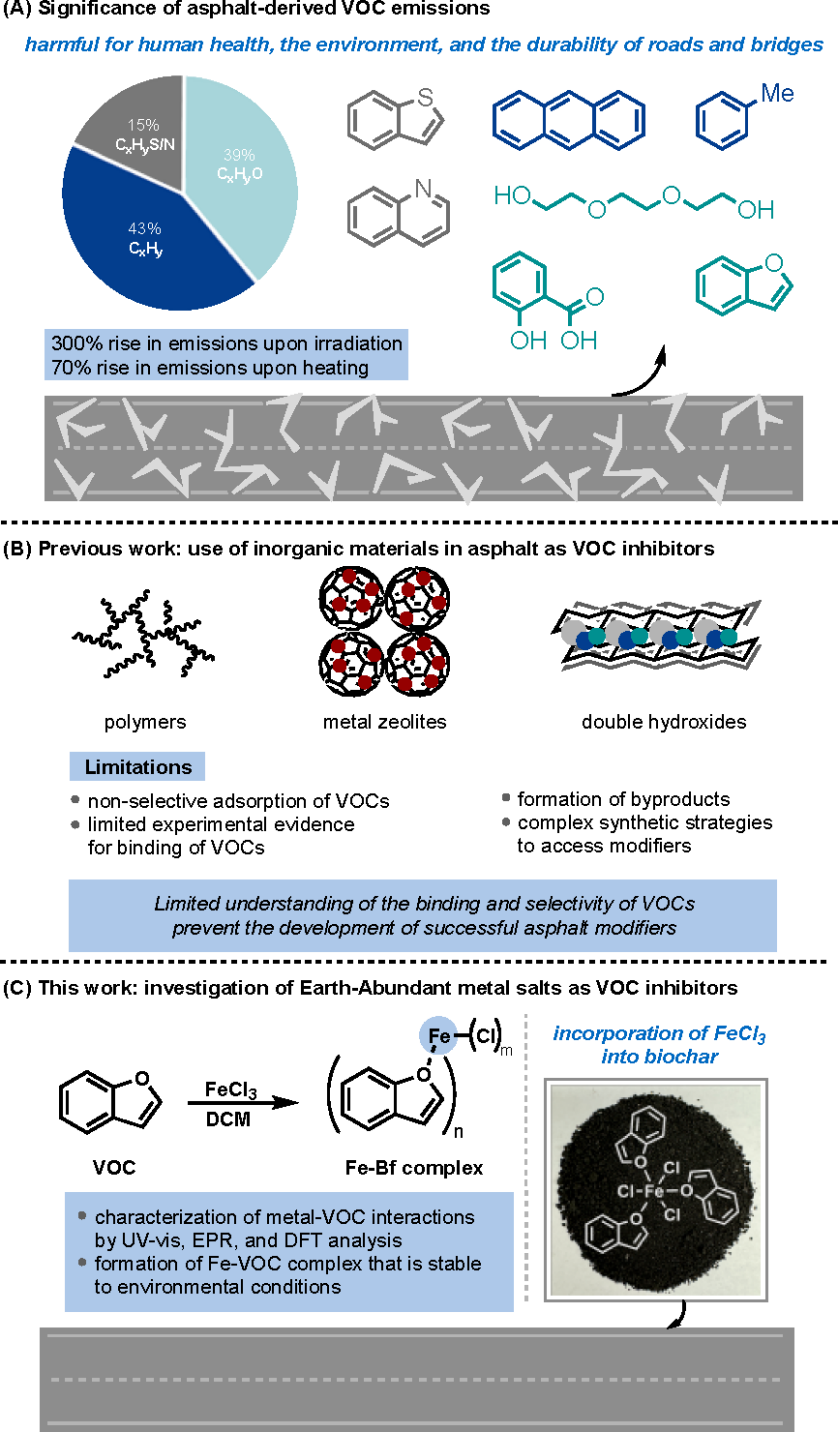
Significance of inhibiting asphalt-derived VOC emissions. (A) Prevalence of VOC emissions upon exposure of asphalt to heat and light. (B) Limitations of the incorporation of inorganic materials in asphalt as VOC inhibitors. (C) Studying the use of Earth-Abundant metal salts in asphalt binder to deter VOC emission.

Volatile organic compounds (VOCs) are organic pollutants that cause significant harm to human health including skin irritation, neurological impairments, respiratory problems, and cardiovascular and reproductive diseases.^1−5^ Due to growing industrialization, increasing amounts of VOCs are being released into the environment from construction sites, commercial products, and vehicle emissions.^6^ Moreover, these compounds contribute to air pollution and are precursors to secondary organic aerosols. Recently, asphalt has been identified as a nontraditional source of VOCs. Small organic molecules such as alkanes, alkenes, aldehydes, aromatics, and heterocyclic compounds have been reported as major constituents of asphalt emissions during the construction and service life of pavement (Figure 1A).^4^ In the United States, more than 2.5 million miles of pavement contribute to VOC emissions.^6^ The loss of VOCs from asphalt increases by 300% upon irradiation and by 70% for every 20 °C rise in temperature.^7−9^ This can also lead to a reduction in the durability of asphalt, further promoting the rate of deterioration of asphalt-surfaced areas.^10^ Studies conducted to evaluate emissions found that asphalt-related VOCs are harmful not only for construction workers but also the public who is frequently exposed to asphalt surfaces.^11−13^ Recently, there has been a significant emphasis on identifying simple and cost-effective solutions for limiting these emissions.^14−16^

The two main strategies proposed to reduce emissions from asphalt-surfaced areas include lowering the construction temperature of hot-mix asphalt and incorporating inhibitors into asphalt that are aimed at trapping and retaining VOCs.^17,18^ While the first strategy is effective during the construction phase, it does not reduce emissions that occur during the service life of asphalt. Therefore, alternative strategies that focus on preventing the degradation of asphalt over time have been explored.^16^ The two categories of VOC inhibitors are cross-linking agents and adsorbents. However, these inhibitors are often limited in suppressing VOCs when they are practically applied to asphalt. In some cases, the inhibitors even reduce the longevity of asphalt and react with the VOCs, leading to the emission of new uncharacterized byproducts.^19−21^ Alternatively, asphalt can be modified using inorganic materials such as metal oxides, salts, and porous substances to reduce VOC emissions without the formation of unintended byproducts. The modification of asphalt is typically achieved heterogeneously with double hydroxides, porous geopolymers, zeolites and other materials that must be carefully formulated prior to use.^22−25^ In contrast, there has been little to no investigation into the direct use of Earth-Abundant metal salts for the adsorption of VOCs that can be added late-stage to the asphalt binder (Figure 1B). Therefore, there is a need to examine scalable and economically viable inorganic salts capable of integrating into asphalt as a modifier that could serve as emission reducing reagents.

Recent work by Fini et al. focused on the selective adsorption of VOCs from asphalt using Fe-rich biochar made from a hybrid feedstock of *Cyanidioschyzon merolae* algae and swine manure (20:80 ratio) with an Fe metal content of 21.8 g/kg. The results indicated that when incorporating Fe-rich biochar into asphalt, up to 76% reduced emissions were observed.^26^ This research finding motivated us to explore the binding capability of Earth-Abundant metal salts like Fe toward VOCs. We began the investigation of Bf as recent studies on the emission of asphalt reported 30% of emissions can consist of oxygen-containing compounds.^8^ Additionally, Bf has been reported by the United States Agency for Toxic Substances and Disease Registry to be a carcinogenic compound whose oral exposure in the range of 60–240 mg kg^–1^ per day was shown by the Centers for Disease Control and Prevention to cause cancer in mice. In this study, we have identified that the incorporation of a simple FeCl_3_ salt into biochar can effectively adsorb Bf (Figure 1C). The interaction between FeCl_3_ and biochar along with the resulting material’s resilience was examined using TGA-DSC analysis, while the coordination of Bf was explored via UV–vis spectroscopy.

## Results and Discussion

### I Investigation of the Binding Activity of Earth-Abundant Metal Salts

**Figure 2:**
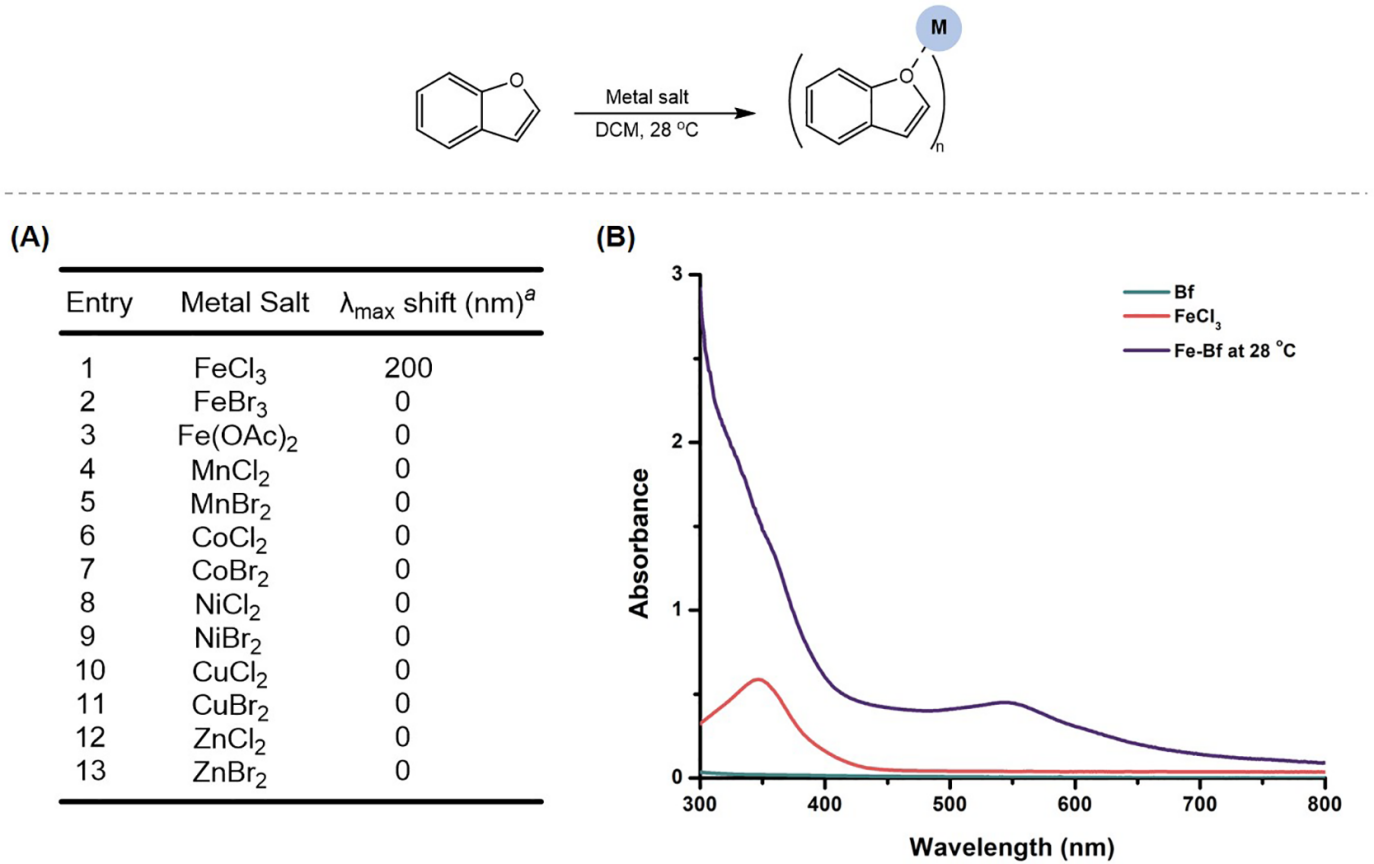
(A) Change in λ_max_ between metal salts with and without Bf. Metal salt (0.05 mmol) and Bf (0.05 mmol) were dissolved in 1 mL of DCM and stirred at 28 °C for 1 h prior to UV–vis analysis. (B) UV–vis spectrum of 200 μM Bf (green), 200 μM FeCl_3_ (red), and a 1:1 mixture of FeCl_3_ and Bf (purple). ^a^Bathochromic shift was observed relative to the absorbance of the metal salt.

To probe the fundamental binding properties of oxygen-containing VOCs, the binding of VOCs to Earth-Abundant metal salts was first investigated. Bf was chosen as a model carcinogenic VOC; it has an aromatic core, Lewis basic oxygen, and a relatively low boiling point.^28^ In addition, Bf has been identified as being emitted from asphalt over a range of temperatures, along with other oxygen- and sulfur-containing VOCs.^9^ To identify metal salts that could effectively bind Bf, and eventually be incorporated into biochar, initial studies were conducted using UV–vis spectroscopy with a noncoordinating solvent system. For each trial, equimolar amounts of the metal salt and Bf were dissolved in 1 mL of dichloromethane (DCM). The solutions were stirred at 28 °C for 1 h prior to UV–vis analysis (Figure 2A). Spectra were examined for bathochromic shifts observed relative to the absorbance of the independent metal salt, suggesting coordination of Bf to the metal. Interestingly, among the 14 Earth-Abundant metal salts studied, only the spectrum collected using FeCl_3_ indicated an interaction with Bf. The appearance of a new red-shifted peak at 554 nm was observed in the spectrum of FeCl_3_ and Bf stirred at room temperature, which could be attributed to an n-π* ligand-to-metal transition (Figure 2B). It was noted that when FeCl_3_ and Bf were mixed in DCM, a deep purple solution was observed that upon evaporation resulted in a purple-colored complex. This is consistent with known Fe complex formation in which FeCl_3_ serves as a strong Lewis Acid.^29^

**Figure 3:**
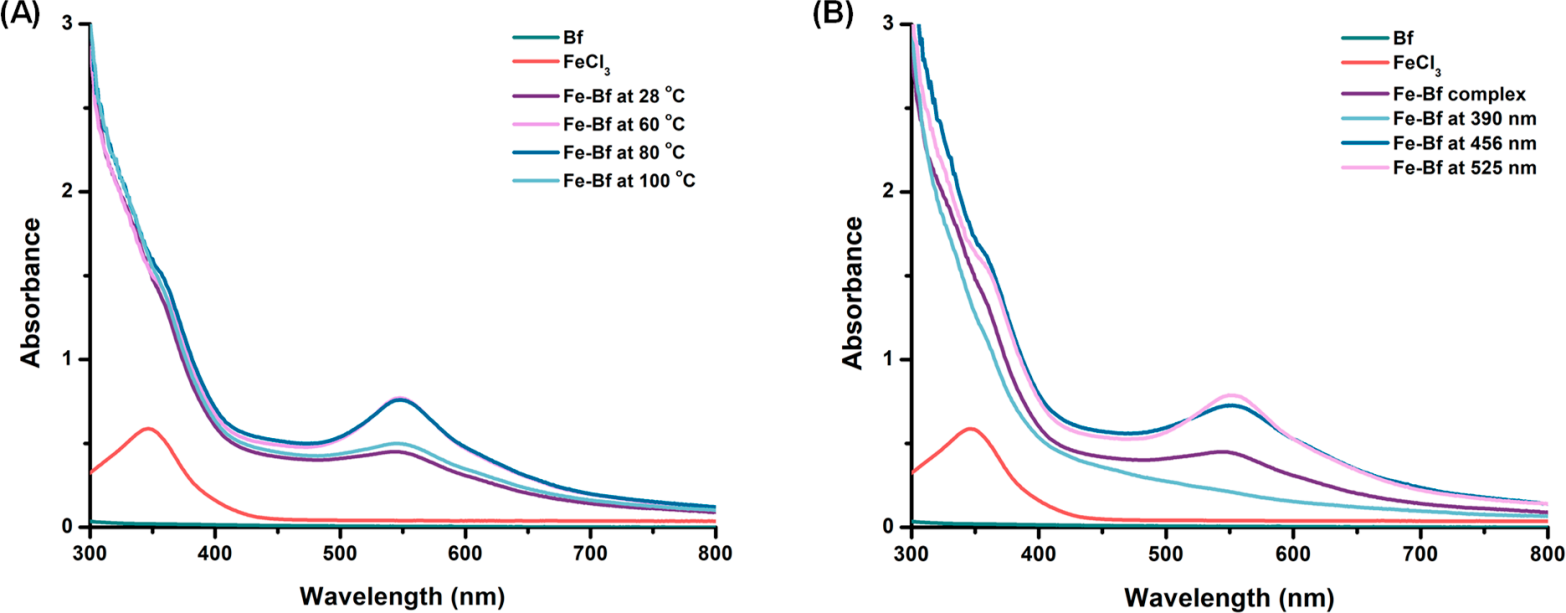
UV–vis spectrum of (A) 200 μM FeCl_3_ (pink), 200 μM Bf (green), and a 1:1 mixture of FeCl_3_ and Bf, stirred for 1 h in DCM in one of four conditions: (i) at 28 °C (purple); (ii) heated at 60 °C (mauve), (iii) 80 °C (dark blue), and (iv) 100 °C (light blue); and (B) 200 μM FeCl_3_ (pink), 200 μM Bf (green), and a 1:1 mixture of FeCl_3_ and Bf, stirred for 1 h in DCM under irradiation conditions at: (i) 390 nm (light blue), (ii) 456 nm (dark blue), and (iii) 525 nm (mauve) wavelengths.

As part of a preliminary assessment of the potential for FeCl_3_ to be an effective metal for direct incorporation into biochar, the effects of temperature and irradiation on Fe-Bf binding were next examined. In prior reports related to asphalt, VOC emissions were observed to be temperature-dependent: as the temperature rises to 60 °C, the amount of emissions doubles, reaching the highest level when the temperature is over 140 °C.^8^ The laying of asphalt is conventionally achieved at 100 °C; therefore it is crucial to investigate the stability of the complex at high temperature.^30,31^ Additionally, upon exposure to light, the emission rate of VOCs accelerates.^8^ Therefore, it is important to investigate the stability of Fe-Bf interactions under these conditions. After the purple complex was formed in DCM (50 mM) by refluxing the metal salt with Bf at 60 °C (Table S1), it was then exposed to elevated temperatures (up to 100 °C). Similarly, the Fe-Bf complex solution was irradiated at different wavelengths (390, 456, and 525 nm), and the stability of the complex was studied in solution phase. The UV–vis absorption spectra were noted for each of the samples and compared with the untreated Fe-Bf complex (Figure 3).

The Fe-Bf complex was stable up to 80 °C, and no degradation was observed upon irradiation at 456 and 525 nm wavelength (Figure 3 and Figure S2). The red-shifted peak was observed at 28 °C, 60 °C, and 80 °C; however, upon further increasing the temperature to 100 °C, the complex began to degrade, as indicated by the diminished intensity of the peak at 554 nm. It is notable that upon heating from 28 to 60 °C, the intensity of the absorbance at 554 nm increased significantly, indicating that mild heating could provide more favorable conditions for the formation of the Fe-Bf complex. The complex remained stable upon irradiation at 456 and 525 nm (visible light) as the absorption peak at 554 nm was unchanged after introduction to light. However, upon irradiation of the mixture at 390 nm (near UV), no peak at 554 nm was observed, suggesting degradation. As the majority of sunlight that is not absorbed by the atmosphere is in the visible range, it is significant that the complex still remained intact when exposed to blue and green light.

#### TGA-FTIR Studies to Support the Binding of Bf to FeCl_3_

**Figure 4:**
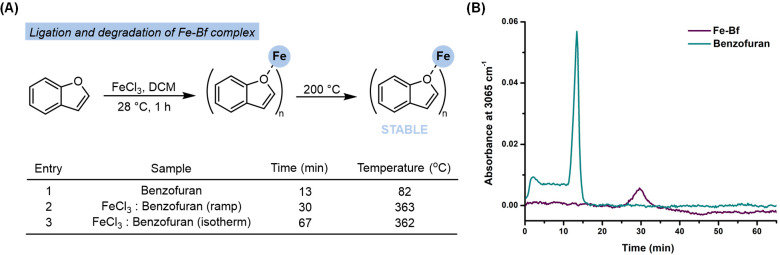
(A) FeCl_3_ (16.2 mg, 0.1 mmol) and Bf (10.8 μL, 0.1 mmol) were mixed with dichloromethane (3.0 mL) at room temperature for 1 h. The solution was then concentrated, and a portion of the sample (16 mg) was transferred to a high-temperature Pt pan for TGA-FTIR analysis. Entry 1 displays a control run for the detection of pure Bf by FTIR at 82 °C upon thermal degradation of 10.8 μL Bf dissolved in 200 μL of DCM analyzed using TGA-FTIR. Entry 2 displays FTIR detection of Bf at 363 °C upon thermal degradation of the Fe-Bf complex using TGA at a ramp of 20 °C/min to 1000 °C. Entry 3 displays FTIR detection of Bf at 363 °C upon thermal degradation of Fe-Bf complex using TGA at a ramp of 20 °C/min to 40 °C followed by an isothermal hold at 40 °C for 10 min to ensure solvent evaporation. This was followed by another ramp at 20 °C/min to 200 °C followed by another isothermal hold for 40 min to assess the thermal stability of the Fe-Bf complex at environmental conditions. Finally, a ramp at 20 °C/min up to 1000 °C was applied to investigate overall thermal stability. (B) Gram-Schmidt profile for Bf (green) and a mixture of FeCl_3_ and Bf samples (purple). The method used included a ramp rate of 20 °C/min to 40 °C, followed by an isotherm hold at 40 °C for 10 min, and ramp rate of 20 °C/min up to 1000 °C.

To further support the formation of the Fe-Bf complex, we used TGA-FTIR analysis to monitor the extrusion of Bf from the Fe-Bf complex through evolved gas IR analysis. The FTIR stretch at 3065 cm^–1^ that corresponds to the Csp^2^–H bond on Bf was examined over time. Upon coordination of Bf, the complex would be expected to have a delayed decomposition time or prolonged degradation at higher temperatures, in comparison to Bf independently. Figure 4A and 4B show the decomposition times and profile curves of the Bf alone and Fe and Bf mixture. Upon analysis by TGA-FTIR pure Bf is detected by FTIR approximately after 13 min at 82 °C. However, when the Fe and Bf mixture was subjected to similar analysis no changes in the IR stretching band at 3065 cm^–1^ were observed until 30 min at 363 °C. The observed delay in the appearance of Bf in the FTIR spectra supports the formation of the Fe-Bf complex. Notably, when this complex was subjected to prolonged heating (at 200 °C for 40 min), which would be expected during the asphalt’s production phase, it remained intact (Figure 4A, entry 3).

### II Characterization of the Fe-Bf Complex

#### Stoichiometry Study of Fe to Bf

**Figure 5:**
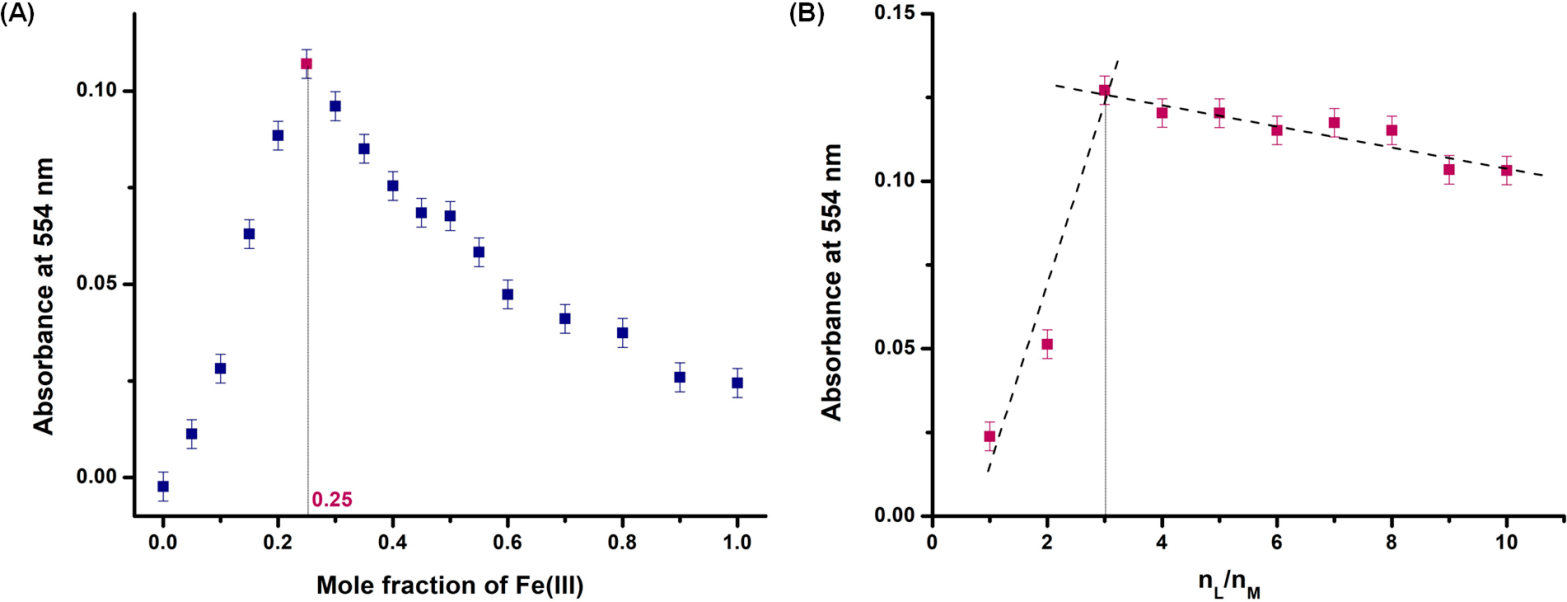
(A) Job plot of the Fe-Bf complex prepared using different mole fractions of Fe(III) and Bf where the total molar concentration of the complex is kept constant; and (B) Molar Ratio plot of the Fe-Bf complex prepared keeping the number of moles of Fe(III) (nM) constant and varying the number of moles of Bf (nL). In both plots, the variations in the absorption intensity of the peak at 554 nm were observed, and the stoichiometry of the Fe-Bf complex was deduced from the maximal position in the Job plot and the highest absorbance in the Molar Ratio plot, suggesting a 1:3 stoichiometry for the Fe-Bf complex.

From UV–vis analysis, it was evident that FeCl_3_ was uniquely capable of binding Bf and that the Fe-Bf complex was stable under elevated temperatures and irradiation. To gain insight into the molecular structure of the Fe-Bf complex, the stoichiometry of the complex was investigated using the Job plot and Molar ratio methods.^32^ In the Job plot method, the Fe-Bf complex was prepared by using different mole fractions of FeCl_3_ and Bf while keeping the total molar concentration of the solution constant (Table S3). UV–vis spectra were collected for the samples, and a plot of the mole fraction of Fe (III) versus the absorbance intensity at 554 nm was obtained. The stoichiometry of the complex was established from the maxima of the Job plot curve (Figure 5A). The nonlinear fitting curve displayed a maximum value at 0.25, suggesting a 1:3 ratio of Fe to Bf for the Fe-Bf complex.^32^ Subsequently, a Molar ratio experiment was conducted. In this study, the number of moles of Bf was varied, keeping the moles of Fe(III) constant in the solution, and the changes in the absorption intensity at 554 nm were noted. The variation in the absorbance intensity of the absorption peak was plotted with respect to the molar ratio of the complex, and the stoichiometry of the complex formed was deduced from the highest absorbance in the plot (Figure 5B).

Furthermore, the strength of the interaction between FeCl_3_ and Bf was calculated using the Benesi–Hildebrand relation (1) and was found to be 0.632 × 10^5^ M^–1^, indicating excellent binding affinity of the metal salt toward Bf, as shown in Figure S6.^33^
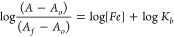
1A_0_, A, and A_f_ are the absorption values in the absence of ferric ion, at the intermediate level of ferric ion concentration, and at the saturation of the ferric ion, respectively. [Fe] is the concentration of the Fe salt in M, and “K_b_” is the binding constant in M^–1^. The stoichiometry studies suggest that the Fe-Bf complex consists of three Bf molecules coordinated to one Fe atom, which may or may not have displaced chlorine atoms. Therefore, subsequent experiments focused on the analysis of the chemical structure by gaining information about the magnetic properties of the Fe-Bf complex.

#### Electron Paramagnetic Resonance (EPR) Spectroscopy

**Figure 6:**
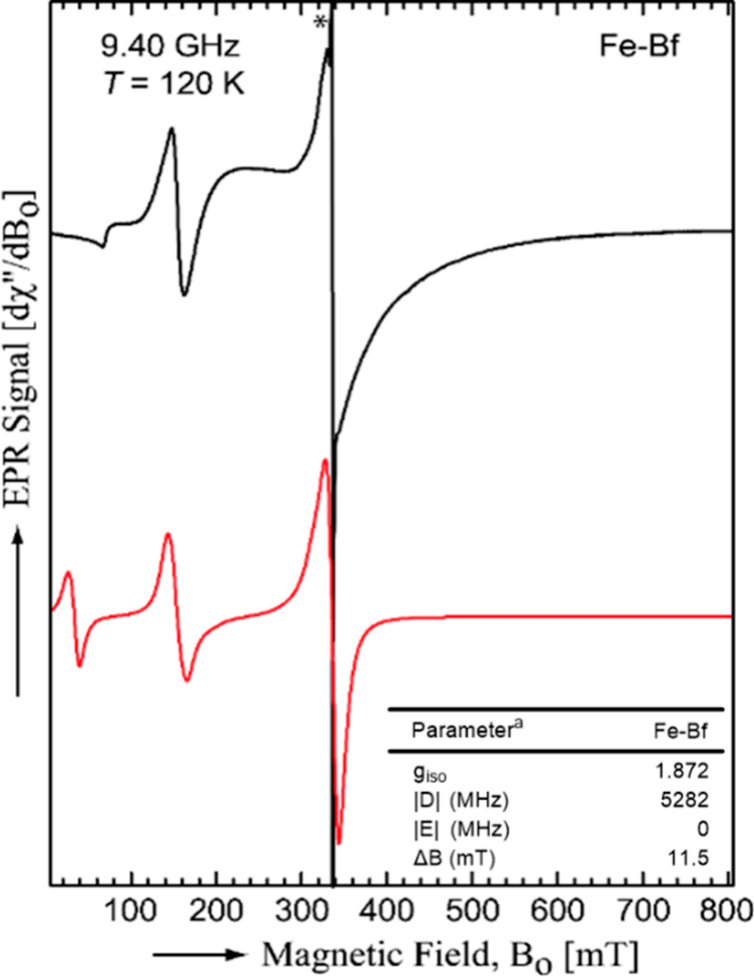
Experimental (black line) and simulated (red line) X-band EPR spectra of Fe-Bf at 120 K. The narrow line around 340 mT (marked with an asterisk) belongs to a minor unidentified species. Inset: parameters used to fit the EPR Spectrum of the Fe-Bf complex at 9.40 GHz and *T* = 120 K. ^a^The fitting parameters were the isotropic g-value (g_iso_), the zero-field splitting parameters (D and E), and the isotropic line width (ΔB).

To study the magnetic behavior of the Fe-Bf complex, the EPR spectrum was collected at 120 K (Figure 6). The X-band (9.40 GHz) EPR spectrum of the Fe-Bf complex (DCM, *T* = 120 K) showed signals consistent with the presence of a single high-spin Fe(III) (black line in Figure 6).^34,35^ To obtain the EPR parameters, the respective spin Hamiltonian was fitted to the data (red line in Figure 6). The observed spectral features were well fit (see Supporting Information) considering an S = 5/2 sextet state with an isotropic *g*-value (*g*_iso_ = 1.872, see inset (Figure 6) for all fitting parameters) and axial zero-field interaction (|*D*| = 5282 MHz and *E* = 0). Furthermore, the value of |*D*| approaches the conditions for resonance at a magnetic field value equal to zero, i.e., |*D*| ≈ ν/2 where ν is the microwave frequency. All these properties indicate an octahedral Fe coordination environment.^35^

Moreover, the best fit of the EPR spectrum was obtained by considering a partially ordered frozen-solution sample. It was considered that most of the complexes are arranged with the planes formed by their equatorial ligands being parallel. Such ordering is probably a consequence of pi stacking among complexes and produces additional broadening of the EPR spectrum (Figure 6). The narrow signal around 340 mT (marked with an asterisk) was not included in the fit, since it belongs to a minor nonidentified species. Coincidentally, its line width (1.2 mT) is like those observed for organic radicals.

#### Magnetic Moment Measurements

To further support the characterization of the Fe-Bf complex the magnetic susceptibility (χ_m_) of the paramagnetic Fe-Bf complex was evaluated using Guoy’s method.^36^ In this experiment, a known amount of the complex was analyzed with [Ni(en)_3_][S_2_O_3_] as a calibrant. The balance reading (R) was used to calculate the gram susceptibility (χ_g_) using the relationship (2) where ‘C’ is the calibration constant calculated using a calibrant whose χ_g_ is known. In this formula ‘L’ is the length of the sample in the sample tube in cm, ‘R’ is the balance reading for the sample, “R_o_” is the balance reading in the absence of the sample, and ‘m’ is the sample mass in g.
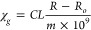
2The value of χ_g_ for [Ni(en)_3_][S_2_O_3_] is 1.103 × 10^–5^ emu/g. After calculating the value of ‘C’, the “χ_g_” for the Fe-Bf complex was calculated to be 4.7931 × 10^–5^ emu/g. Subsequently, the molar susceptibility (χ_m_) was calculated using eq 3 where ‘M’ is the molecular weight of the complex in g/mol.
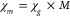
3Since the molecular weight of the complex was unknown, the value of “χ_m_” was obtained from the molecular weight of the possible Fe-Bf structures that could be formed with a 1:3 ratio of FeCl_3_ to Bf.

**Figure 7:**
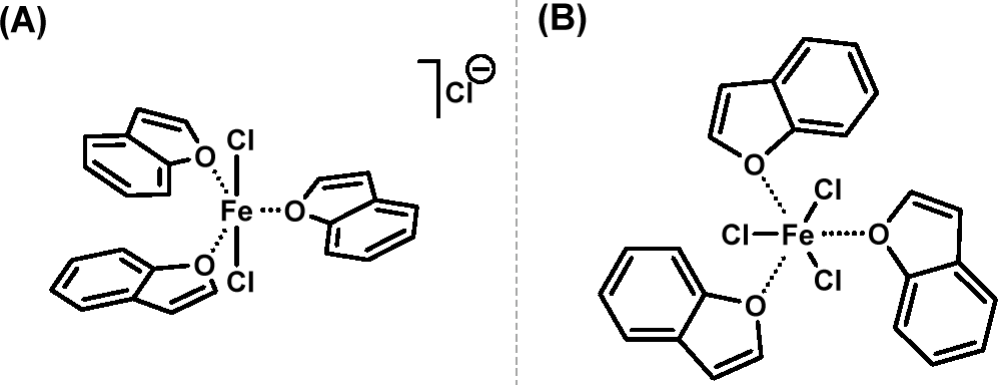
Proposed chemical structures of the high-spin Fe-Bf complex. (A) [Fe(Bf)_3_Cl_2_]Cl^–^ and (B) Fe(Bf)_3_Cl_3_ based on EPR and magnetic susceptibility results.

χ_m_ is in turn related to the effective magnetic moment (μ_eff_) by the relationship (4) and μ_eff_ can be used to calculate the number of unpaired electrons (n) present in a paramagnetic complex using (5).
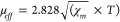
4
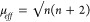
5It was deduced from the calculations that the possibility of the Fe atom to exist in low-spin conditions is negligible, which is also supported by the experimental findings using EPR analysis. The value of μ_eff_ was calculated for the following possible geometries of the Fe-Bf complex (Figure 7). The molecular weight of the structure is 516.60 g/mol, and the value of χ_m_ was calculated to be 0.01451801 emu/mol. From the calculated χ_m_, the μ_eff_ was obtained to be 5.88 μ_B_ which is close to the experimental value of 5.9 μ_B_. Relationship (5) was then employed to calculate the number of unpaired electrons which was found to be 4.97.

The spectroscopic analysis indicated a stoichiometry of 1:3 Fe/Bf in the complex. EPR analysis as well as magnetic susceptibility calculations suggested the presence of a high-spin Fe(III) center in the complex structure and supported the plausibility of the complex to exist in either an octahedral or trigonal bipyramidal geometry. In addition, the EPR analysis indicated that it is highly unlikely that the complex would have two metal centers. Therefore, based on the spectroscopic, magnetic susceptibility, and EPR results, the following two geometries of the Fe-Bf complex were proposed in Figure 7.

### III DFT Modeling

**Figure 8:**
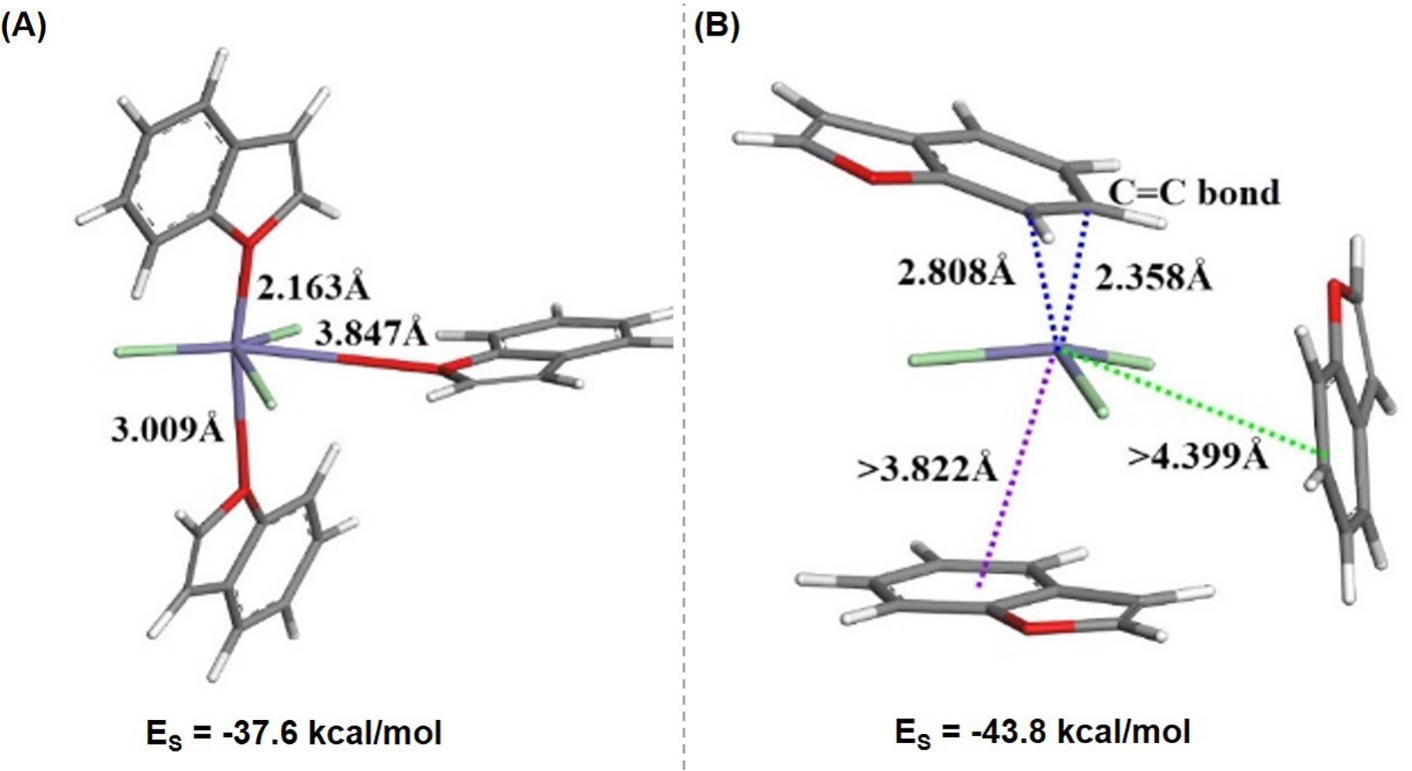
Optimized geometries of the Fe(Bf)_3_Cl_3_ complexes displaying bond length (Å) of Fe–O bond and Fe–C bond and stabilization energy (E_S_) of the two optimized geometries in kcal/mol. Atom colors are Fe-violet, Cl-green, O-red, C-gray, and H-white.

To assess the intermolecular interactions between FeCl_3_ and Bf, four methods of interaction were probed, depicted in eqs 6–9. The geometries of the species involved in the reactions were optimized by using an implicit continuum solvation model, COSMO. Stabilization energy (E_S_) for these interactions were calculated using eq 1 (Section I in the Supporting Information). Equation 6 demonstrates the addition of three Bf molecules to FeCl_3_ while the three chlorine atoms remain attached to the metal center. Equations 7–9 illustrate the formation of Fe-Bf ions with three, two, and one Bf molecule(s), respectively. Results indicate that separation of the Cl atom from the iron center and formation of the complex ions in a nonaqueous medium (DCM solvent with a dielectric constant of 9.08) was not thermodynamically favorable. These findings were supported by positive E_S_ values of 212.8, 130.1, and 56.6 kcal/mol for eq 7, eq 8, and eq 9, respectively. Moreover, a significant and negative value of E_s_ was detected for the formation of the neutral Fe(Bf)_3_Cl_3_ complex (eq 6). Two distinct stable geometries were detected, as shown in Figure 8. In one complex, the iron center interacted with the oxygen atoms of the Bf molecules (Figure 8A). The other complex showed the π-electron cloud of the benzene rings of the Bf molecules interacting with the iron center (Figure 8B).
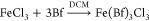
6
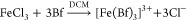
7
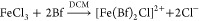
8
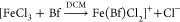
9

### IV Selectivity Study of the Complex

Literature precedent has demonstrated that asphalt can be a source of a wide range of VOC emissions containing anthracene, fluoranthene, fluorene, 1-methyl naphthalene, benzothiophene, dibenzothiophene and other oxygen-, nitrogen-, and sulfur-containing organic molecules.^37^ The complexity of the composition as well as the emissions of asphalt fumes increase with age, usage, temperature, and UV exposure. Therefore, to develop a robust system that can efficiently suppress the emission of these hazardous compounds, the selectivity of the material in the presence of different VOCs and different concentrations is crucial. To address this need, we investigated the stability of the Fe-Bf complex upon being subjected to different VOCs that have been reported in the literature. To gain insight into the possible interactions between the competing VOCs and the Fe-Bf complex, the Fe-Bf complex was examined in the presence of different VOCs by using UV–vis spectroscopy. Control samples of FeCl_3_ with different VOCs were also independently analyzed to rule out the possibility of new observed absorption bands resulting from electronic transitions of the VOC molecule.

**Figure 9:**
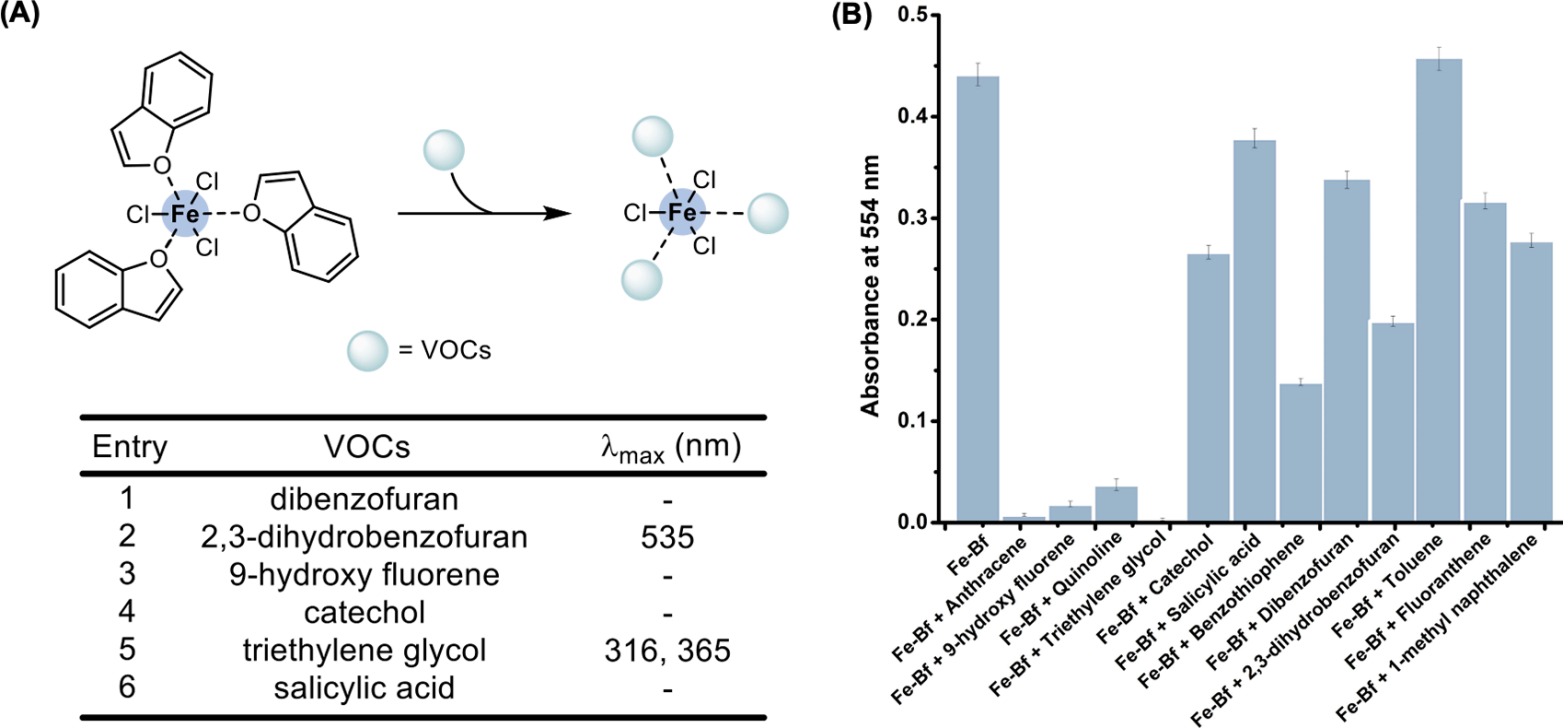
(A) Schematic representation of the binding activity of the complex with Oxygen-containing VOCs evaluated using UV–vis absorption spectroscopy displaying distinct absorption peaks (λ_max_) summarized in tabular form. *Note*: FeCl_3_ (0.1 mmol), Bf (0.3 mmol), and VOC (0.3 mmol) were dissolved in DCM (2 mL). The solutions were stirred at 60 °C for 1 h prior to UV–vis analysis. Note: ‘-’ indicates that no spectroscopic changes were observed. (B) Bar graph displaying variation in the intensity of the absorption peak at 554 nm (characteristic of the Fe-Bf complex) in the presence of different VOCs. The UV–vis absorption peak was observed after subjecting the Fe-Bf complex (50 mM) to 0.3 mmol of VOCs in DCM and diluting it before recording the spectra.

**Figure 10:**
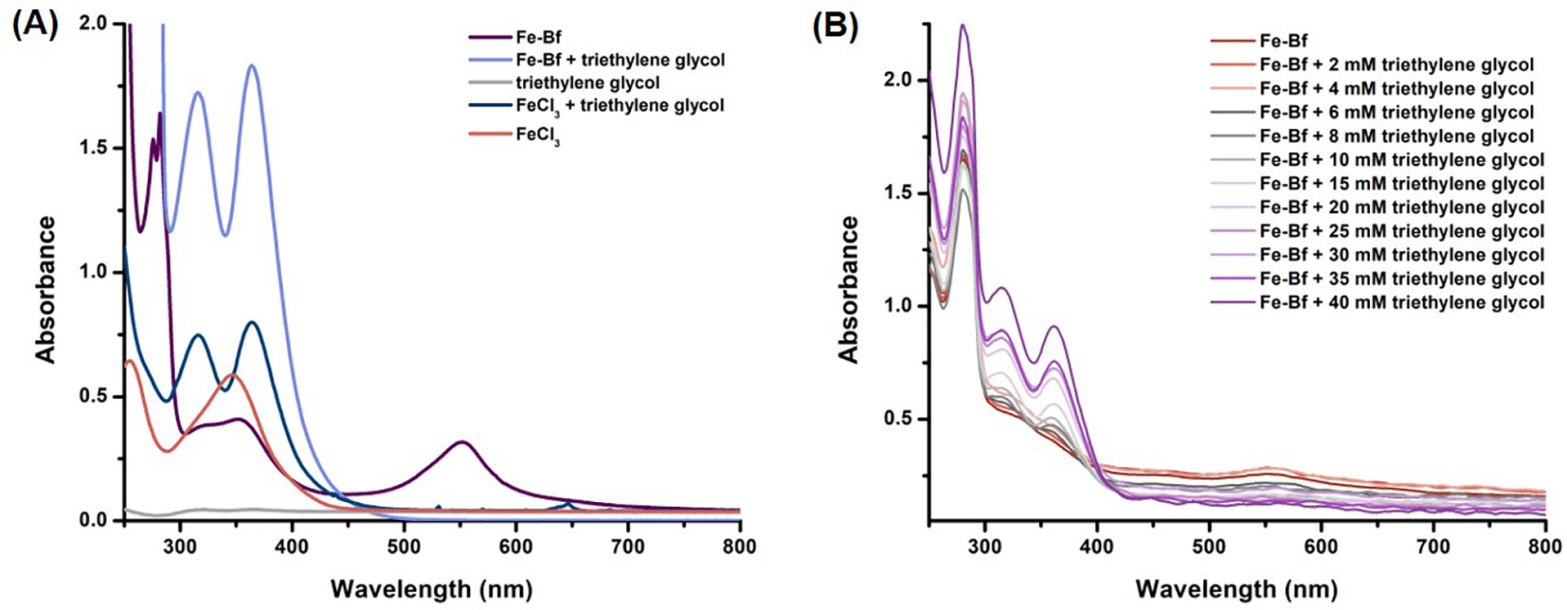
(A) UV–vis absorption spectra of the Fe-Bf complex in the presence of triethylene glycol, along with FeCl_3_, FeCl_3_-triethylene glycol, triethylene glycol, and the Fe-Bf complex as controls under the same conditions. (B) UV–vis absorption spectra of the Fe-Bf complex in the presence of increasing concentrations of triethylene glycol (0–40 mM), displaying distinct absorption peaks (316 and 365 nm) along with the disappearance of the absorption peak at 554 nm.

First, the Fe-Bf complex was tested in the presence of different oxygen-containing VOCs. We examined the UV–vis spectra of the complex in the presence of 0.3 mmol of 2,3-dihydrobenzofuran (2,3-DHB), 9-hydroxy fluorene, catechol, triethylene glycol (TEG) and salicylic acid in DCM (1.0 mL). Among the studied VOCs, only 2,3-DHB and TEG displayed an interaction with the complex (Figures 9A, S7, and S10C). In the presence of 2,3-DHB, a new distinct absorption peak at 535 nm was observed and the absorption peak at 554 nm diminished. The interaction between the Fe-Bf complex and 2,3-DHB was investigated by slowly subjecting the preformed complex to small aliquots of 2,3-DHB and monitoring the absorption spectrum after each addition (Figure S12). It was inferred from the absorption spectrum that 2,3-DHB could destabilize the Fe-Bf complex, as the intensity of the peak at 554 nm decreased. Alternatively, when the Fe-Bf complex was subjected to TEG, distinct absorption bands at 316 and 365 nm were observed (Figure 9A). Analogously, a titration experiment conducted in the presence of TEG showed that the complex was gradually destabilized with no Fe-Bf complex detected after the addition of 40 mM TEG (Figure 10B). This aligned with our molecular modeling calculations showing that the TEG molecule could substitute for the Bf ligand in the Fe-Bf complex (Figure 12B).

**Figure 11:**
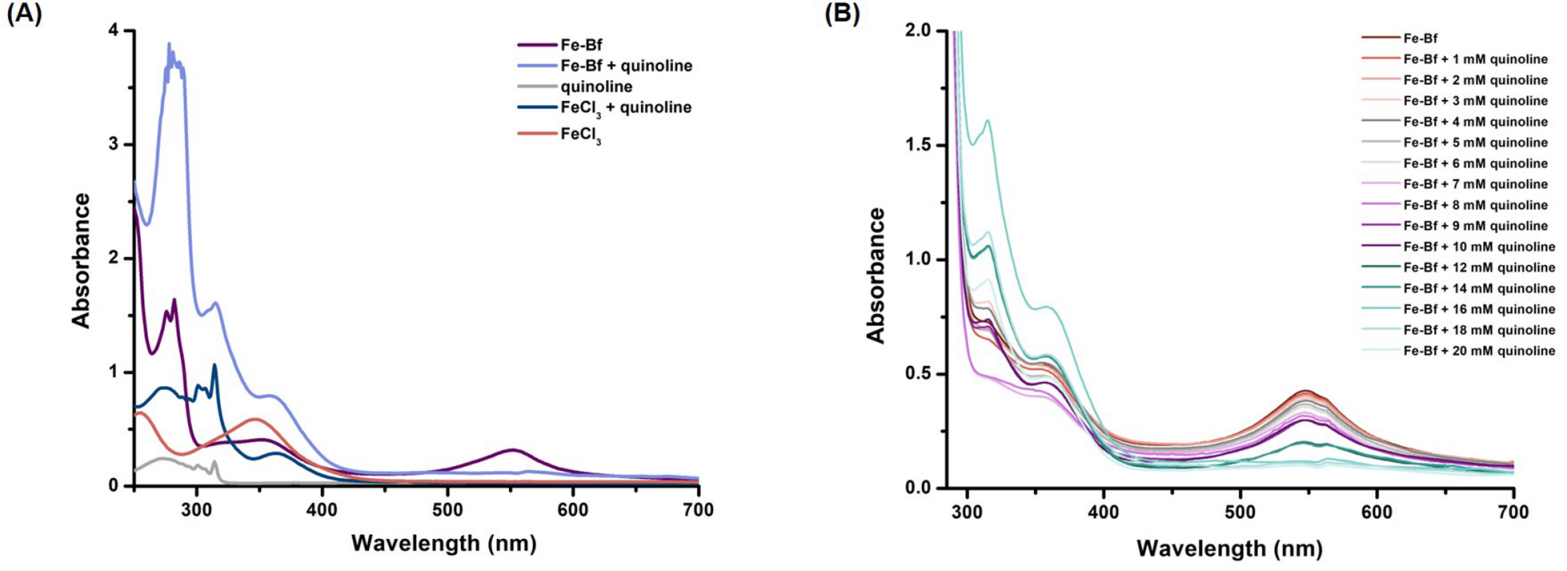
(A) UV–vis absorption spectra of the Fe-Bf complex in the presence of quinoline, along with FeCl_3_, FeCl_3_-quinoline, quinoline, and the Fe-Bf complex as controls under the same conditions. (B) UV–vis absorption spectra of the Fe-Bf complex in the presence of increasing concentrations of quinoline (0–20 mM) displayed distinct absorption peaks along with the disappearance of the absorption peak at 554 nm.

**Figure 12:**
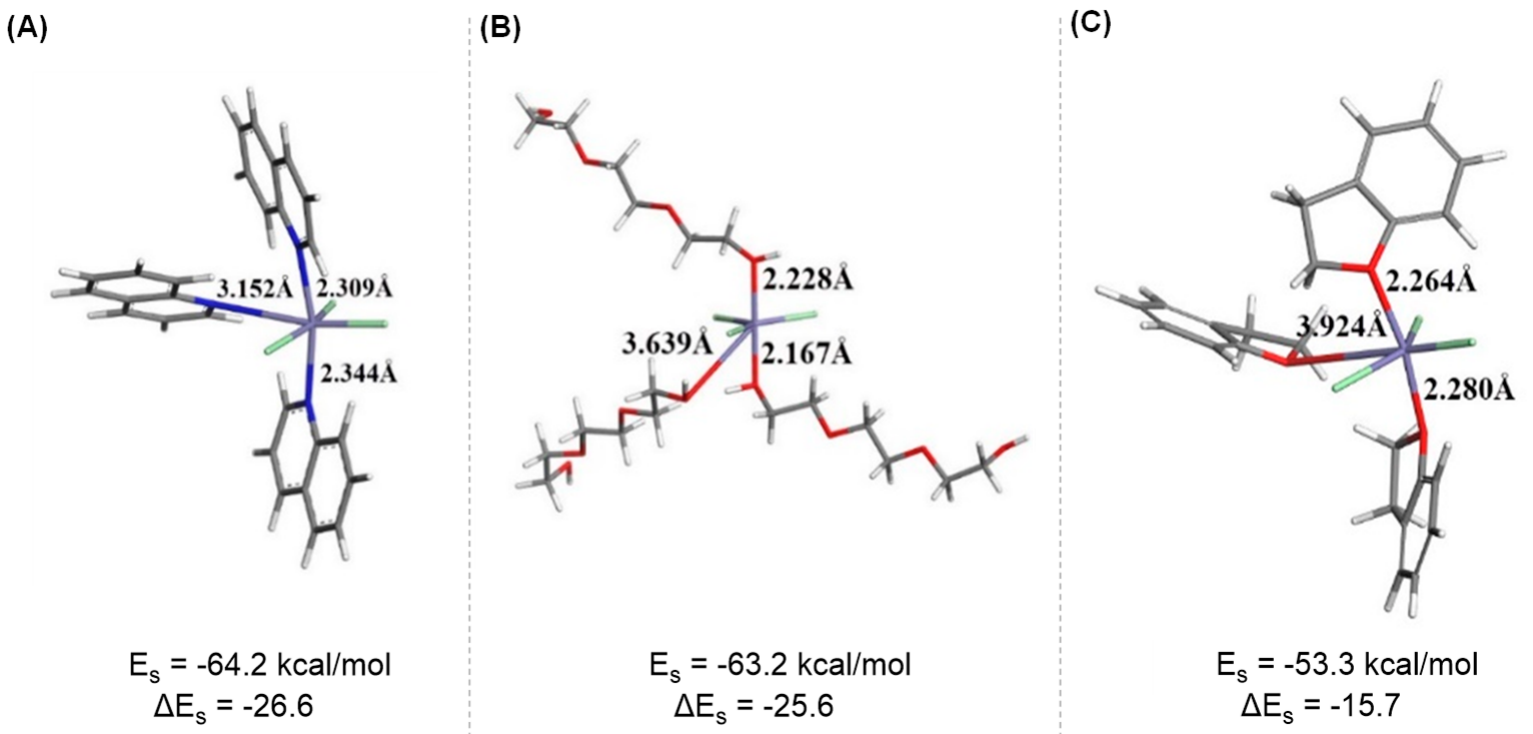
Optimized geometries *E*_S_ and Δ*E*_S_ for the formation of (A) Fe(quinoline)_3_Cl_3_, (B) Fe(TEG)_3_Cl_3_, and (C) Fe(2,3-DHB)_3_Cl_3_. Atom colors are Fe-violet, Cl-green, O-red, N-blue, C-gray, and H-white.

We further investigated the stability of the Fe-Bf complex in the presence of benzothiophene, a sulfur-containing VOC that is emitted from asphalt. The solution containing the complex and benzothiophene was analyzed by using UV–visible spectroscopy to observe any spectroscopic changes. The absorption spectrum displayed no characteristic peak in the UV–vis region, suggesting no interaction between Fe(III) and benzothiophene (Figure S10A). The stability of the complex in the presence of other VOCs such as 1-methyl naphthalene, quinoline, anthracene, fluoranthene, and toluene was also investigated. The complex was subjected to 0.3 mmol of these VOCs and analyzed spectrophotometrically. Among these analyses, quinoline was found to destabilize the Fe-Bf complex, and a new peak at 365 nm was observed, indicating an interaction between FeCl_3_ and quinoline (Figure 9B and Figure S8). The selectivity results are summarized in Table S4. Interestingly, no new peaks were observed in the presence of anthracene. This could indicate that anthracene disrupts the complexation of the Fe-Bf species without the formation of any new species. The interaction between the Fe-Bf complex and quinoline was further studied by a titration study. This demonstrated that upon increasing the concentration of quinoline, the Fe-Bf complex was gradually destabilized and was not observed after the addition of 20 mM quinoline (Figure 11B). Our computational modeling using density functional theory supported the higher stability of the Fe-quinoline complex, which is explained in greater detail in the DFT section (Figure 12A).

### V DFT Modeling for Evaluation of the Stable Fe-VOC Complexes

2,3-DHB, TEG and quinoline were the three VOCs that could displace the Bf ligand in the Fe-Bf complex. To assess the stability of the Fe(Bf)_3_Cl_3_ complex in the presence of these VOCs, chemical reactions 10 and 11 were considered. In cases where different VOC orientations were possible, the structure with the maximal stabilization energy was reported. All of these VOC molecules have oxygen or nitrogen heteroatoms in their structures that can form a complex similar to that shown in Figure 12A. To analyze the stability of the new Fe-VOC complexes, their E_S_ values were calculated for the formation of Fe-VOC complexes from an FeCl_3_ molecule (eq 10) and the Δ*E*_S_ for the formation of Fe-VOC complexes from the Fe(Bf)_3_Cl_3_ complex (eq 11).
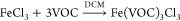
10
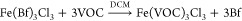
11

DFT results indicated that these VOCs formed new complexes with FeCl_3_ that were more stable than the Fe(Bf)_3_Cl_3_ complex and consequently could replace the Bf ligands in Fe(Bf)_3_Cl_3_. The geometries of these new complexes and their *E*_S_ and Δ*E*_S_ are presented in Figure 12. The higher stability of these new complexes was reflected in the Δ*E*_S_ values that were −26.6 kcal/mol, −25.6 kcal/mol, and −15.7 kcal/mol for interactions of quinoline, TEG, and 2,3-DHB, respectively. Alongside the Δ*E*_S_ values, the optimized geometries showed shorter distances between the iron center and heteroatoms of these VOCs compared to those of Bf, indicating stronger coordination between iron and the heteroatoms of these ligands.

### VI Direct Application of Fe-Incorporated Biochar to Bf Inhibition

The interaction between Bf and FeCl_3_ to form a stable Fe(Bf)_3_Cl_3_ complex motivated us to apply the metal salt as a late-stage dopant in an asphalt modifier to reduce Bf emissions. Since Fe-rich biochar has precedent in reducing VOC emissions up to 76%, we focused on utilizing algae biochar. Algae biochar was treated with FeCl_3_ (Section III, Supporting Information) and analyzed spectroscopically. It was observed from the UV–vis spectra that a new shoulder peak appeared at 405 nm with the treated biochar, which was not a characteristic peak of pristine biochar or FeCl_3_ independently (Figure S13). The treated biochar was also analyzed using FTIR spectroscopy to confirm the incorporation of the Fe salt into biochar, and the spectra displayed a few defined vibrational stretches at 1020–1160 and 1590 cm^–1^ in addition to the loss of characteristic IR stretching and bending vibrations of FeCl_3_·6H_2_O as shown in Figure S14A. Both pieces of data indicated that Fe had been altered by association with the biochar. With evidence for Fe incorporation into biochar, the adsorption capacity of the biochar for Fe(III) was then calculated to be 13.02 mg/g. To obtain the adsorption capacity, a spectroscopic calibration plot of the known concentrations of the metal salt was obtained (Figure S15) and the amount of Fe(III) remaining was calculated in the filtrate after the treated biochar. Next, the thermal stability of the treated biochar was examined to ensure that the biochar does not degrade with the addition of Fe salt. The TGA and DTG curves of the biochar after treatment with FeCl_3_ displayed a robust material in comparison to the pristine biochar (Figure S14B and Figure S16). After validating the incorporation of Fe(III) into biochar and investigating the stability of the material thermogravimetrically, the treated biochar’s binding capability to Bf was tested via spectroscopic analysis. The modified biochar was solubilized in DCM and subjected to 3 equiv of Bf (with respect to adsorbed Fe(III) concentration calculated using a calibration plot) and stirred at 60 °C for 1 h. The complex formed displayed a sharp absorption band at 554 nm (Figure S13) consistent with the stable Fe(Bf)_3_Cl_3_ complex. This result confirmed that Fe-incorporated biochar can be employed to suppress Bf emissions. Additionally, this simple procedure for identifying and incorporating Earth-Abundant metal salts into modifiers could be broadly applicable in sustainable engineering.

## Conclusion

Asphalt is a nontraditional source of pollutants that adversely affects air quality and public health. To investigate sustainable and cost-effective asphalt materials that can retain VOCs under environmentally relevant conditions, we examined the use of Earth-Abundant metal salt dopants. It was found that FeCl_3_ was a promising candidate for binding a selection of VOCs using UV–vis and TGA-FTIR spectroscopy. The formation of an octahedral Fe(Bf)_3_Cl_3_ complex when FeCl_3_ was in the presence of an excess of Bf was supported by stoichiometry studies, DFT, EPR, and magnetic moment analyses. The Fe-Bf complex was determined to be thermally stable up to 360 °C and resistant to degradation upon exposure to visible light. Selectivity studies between distinct VOCs in a homogeneous medium demonstrated that once Bf coordinated to the Fe salt, the complex was stable even in the presence of 1-methyl naphthalene, fluoranthene, anthracene, 9-hydroxy fluorene, catechol, salicylic acid, and benzothiophene. However, nitrogen-containing VOCs such as quinoline and oxygen-containing VOCs such as triethylene glycol and 2,3-dihydrobenzofuran were able to successfully replace Bf and form new Fe coordination complexes. The feasibility of applying this promising dopant to asphalt was demonstrated when FeCl_3_ was successfully adsorbed on Algal Biochar, which is a known asphalt binder. The Fe-enriched Biochar exhibited thermal stability by TGA-DSC analysis. Additionally, Fe-biochar was shown to bind Bf, exhibiting the same spectroscopic signatures characteristic of Fe(Bf)_3_Cl_3_. The present study is promising for the development of sustainable asphalt engineering where asphalt modifiers with the late-stage addition of inexpensive Earth-Abundant metal salts can be used to combat VOC emissions.

## Supporting Information Available

This includes the following: general information about reagents, solvents, and instrumentation as well as procedures for experimentation and analysis (UV–vis,TGA-FTIR, TGA-DSC, EPR, and DFT studies (PDF)
