# Supplementary material for: Investigation of Earth-Abundant Metal Salts for the Inhibition of Asphalt-Derived Volatile Organic Compounds
Source: ACS Omega. 2024 May 13;9(21):22941–51. doi: 10.1021/acsomega.4c02095 (PMC11137726; doi:10.1021/acsomega.4c02095)
Supplement: Supplementary file 1 — ao4c02095_si_001.pdf [file ao4c02095_si_001.pdf]

Investigation of Earth-Abundant Metal Salts for the Inhibition of Asphalt-Derived Volatile Organic Compounds

Harpreet Kaur, Reem Nsouli, Gabriella Cerna, Saba Shariati, Marco Flores, Elham H. Fini,*
Laura K. G. Ackerman-Biegasiewicz*

Department of Chemistry, Emory University, Atlanta, GA 30322
School of Molecular Sciences, Arizona State University, 660 S. College Avenue, Tempe, AZ 85287-3005, USA
School of Sustainable Engineering and the Built Environment, Arizona State University, 660 S. College Avenue, Tempe, AZ 85287-3005, USA

Supporting Information

CONTENTS

I. GENERAL INFORMATION……………………………………...………………..…….…….…….….…….3

II. EXPERIMENTAL PROCEDURES.…….........................……………………………….….………….……4

A. General procedure for the synthesis of the Fe-Bf complex
B. General procedure for the magnetic moment measurement of the Fe-Bf complex

III. UV-vis SPECTROSCOPY STUDIES……………………….…………………..….…….….……….5

A. General procedure for the study of the interaction between metal salts and Bf
B. General procedure for the study of the stability of the Fe-Bf complex upon heating
C. General procedure for the study of the stability of the Fe-Bf complex upon irradiation
D. General procedure for the study of the stability of the Fe-Bf complex in different solvents
E. General procedure for the study of the formation of the Fe-Bf complex at different stoichiometric ratios of Fe and Bf
F. General procedure for the molar ratio method and Job plot
G. General procedure for the evaluation of the binding constant for the complex using the Benesi-Hildebrand relation
H. General procedure for the study of the selectivity of $FeCl_3$ in the presence of other VOCs
I. General procedure for the direct application of Fe-incorporated biochar for Bf inhibition

IV. TGA-FTIR STUDIES……………………………………………….……………………………..9

A. Procedure to confirm binding of Bf to $FeCl_3$
B. Procedure for thermal analysis of the degradation of the Fe-Bf complex

V. SPECTROSCOPIC DATA.……………………………………………………………..…………...9

A. Stability of the Fe-Bf complex at different temperatures and wavelengths of irradiation
B. Stability of the Fe-Bf complex in DCM/methanol system established using UV-vis spectroscopy
C. Benesi-Hildebrand plot
D. Selectivity of $FeCl_3$ in binding different VOCs and the Fe-Bf complex upon addition of different VOCs
E. Direct application of Fe-incorporated biochar for Bf inhibition

I. General Information

MATERIALS

Unless otherwise stated, all reagents and substrates are commercial, purchased as reagent grade, and used without further purification. Materials were sourced from Sigma-Aldrich, Oakwood Chemical, J.T. Baker, and Combi-Blocks. All metal salts were obtained from Oakwood Chemical. The VOCs under study were obtained from either Combi-Blocks or Oakwood Chemical. HPLC-grade dichloromethane (DCM) was obtained from J.T. Baker.

INSTRUMENTATION

Ultraviolet-Visible (UV-vis) absorbance spectra were collected on an Agilent Cary 60 UV-vis spectrophotometer using a quartz cuvette with a pathlength of 1 cm. Thermogravimetric analysis (TGA) was performed using a TA TGA5500 discovery series instrument (5500-0528) and high temperature 100 µL platinum pans (T 190613; 957571.901) for investigating thermal stability of Fe-Bf complex. To assess the thermal stability of pristine and treated biochar, TGA was performed on Mettler Toledo TGA-DSC 3+ system using 70 µL Al_2O_3 pan. Fourier transform infrared (FTIR) spectra were recorded on a Thermo-Fischer Nicolet iS50 and are reported in terms of frequency of absorption (cm^{-1}). Electron Paramagnetic Resonance (EPR) spectroscopic studies were performed at the EPR facility of Arizona State University. Continuous wave (CW) EPR spectra were recorded at 120 K using a Bruker ELEXSYS E580 CW X-band spectrometer (Bruker, Rheinstetten, Germany) equipped with a liquid nitrogen temperature control system (ER 4131VT). The magnetic field modulation frequency was 100 kHz with a field modulation amplitude of 1 mT peak-to-peak. The microwave power was 1 mW, the microwave frequency was 9.40 GHz, and the sweep time was 168 s. The magnetic susceptibility of the complex was measured by Gouy's balance using $[Ni(en)_3][S_2O_3]$ as a calibrant (Johnson Matthey, USA).

COMPUTATIONAL METHOD

The interaction of $FeCl_3$ with Bf and candidate VOCs was examined using DFT. For this purpose, the Dmol3 module implemented in the Accelrys Materials Studio program package (Version 7) was used.[1] All geometries involved in the interactions were optimized using Perdew−Burke−Ernzerhof (PBE) exchange-correlation functional with Grimme's dispersion correction (PBE–D) and all-electron double-numerical polarized basis set (DNP).[2-3] Optimizations were performed with the convergence criteria of 2.0×10^{-5} Eh, 4.0×10^{-3} Eh/Å, and 5.0×10^{-3} Å for energy, maximum force, and displacement, respectively. Calculations were attained in a solvent medium and the conductor-like screening model (COSMO) was used to account for solvent effects considering DCM as the solvent with the dielectric constant of 9.08.[4-5] Stabilization energies (E_S) for formation of complexes were evaluated using Eq. S1. The electrostatic COSMO potential was integrated into the SCF procedure, so the optimized total energies of the product comprised solvent effects.

$$E_S = \Sigma E_{t\ (products)} - \Sigma E_{t\ (substrates)} \qquad (S1)$$

In the above formulation, $E_{t\ (products)}$ is the total energy of the products, and $E_{t\ (substrates)}$ is the total energy of the substrates in a specific reaction. Negative values of E_S indicate the thermodynamic stability of the products.

ELECTRON PARAMAGNETIC RESONANCE SPECTROSCOPY

Spin Hamiltonian. The EPR spectrum of the Fe compound was interpreted using a S = 5/2 spin Hamiltonian, *H*, containing the electron Zeeman interaction with the applied magnetic field $\mathbf{B_0}$, and the zero-field interaction.[6]

$$H = \beta_e\, \mathbf{S} \cdot \boldsymbol{g} \cdot \mathbf{B_0} + h\, \mathbf{S} \cdot \boldsymbol{D} \cdot \mathbf{S} \qquad (S2)$$

Where, '**S**' is the electron spin operator, '***D***' is the zero-field interaction tensor in frequency units, '***g***' is the electronic *g*-tensor, 'β_e' is the electron magneton, and '*h*' is Planck's constant.

Fitting of EPR Spectra. To quantitatively compare experimental and simulated spectra, we divided the spectra into N intervals. We treated the spectrum as an N-dimensional vector **R**. Each component R_j has the amplitude of the EPR signal at a magnetic field B_j with j varying from 1 to N. The amplitudes of the experimental and simulated spectra were normalized so that the span between the maximum and minimum values of R_j was 1. We compared the calculated amplitudes R_j^{calc} of the signal with the observed values R_j defining a root-mean-square deviation 'σ' by:

$$\sigma(p_1, p_2,\ldots, p_n) = [\sum_j (R_j^{calc}(p_1, p_2, \ldots, p_n) - R_j^{exp})^2/N]^{½} \qquad (S3)$$

where the sums are over the N values of j, and p's are the fitting parameters that produced the calculated spectrum. For our simulations, N was set equal to 2048. The EPR spectrum was simulated using EasySpin (v 5.2.35), a computational

package developed by Stoll and Schweiger and based on Matlab (The MathWorks, Natick, MA, USA).[7] EasySpin calculates EPR resonance fields using the energies of the states of the spin system obtained by direct diagonalization of the spin Hamiltonian (Eq. S2). The EPR fitting procedure used a Monte Carlo type iteration to minimize the root-mean-square deviation, σ (Eq. S3), between measured and simulated spectra. We searched for the optimum values of the following parameters: the isotropic g-value (g_{iso}), the zero-field splitting parameters (D and E), and the isotropic peak-to-peak line width (ΔB).

II. Experimental Procedures

A. GENERAL PROCEDURE FOR THE SYNTHESIS OF THE Fe-Bf COMPLEX:

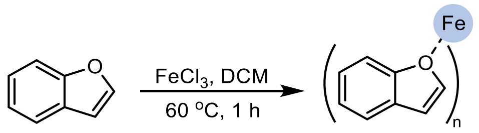

A 1-dram vial (VWR glass vials, 470151-622) equipped with a PTFE-coated stir bar (VWR spinbar micro, 3 x 10 mm, 58948-375) was charged with DCM (2.00 mL). To the reaction vial $FeCl_3$ (0.10 mmol) and Bf (0.30 mmol) were added. The vial was fitted with a cap and the reaction was stirred at 300 rpm for 1 h at 60 °C. After heating, the reaction was filtered using filter paper placed in a glass funnel and the filtrate was concentrated in vacuo. Unless otherwise stated, this procedure was used for the synthesis of the complex used for the stability, stoichiometry, and selectivity studies (Section III-Section V). For the synthesis of the Fe-Bf complex and other experiments described below, no unexpected or unusually high safety hazards were encountered.

B. GENERAL PROCEDURE FOR THE MAGNETIC MOMENT MEASUREMENT OF THE Fe-Bf COMPLEX:

The magnetic properties of the Fe-Bf complex were investigated to understand the paramagnetic susceptibility (χ_M) of the paramagnetic Fe-Bf complex using EPR spectroscopy and Guoy's method. In the EPR experiment the tube was filled with 500 µL DCM containing 5 mg of the Fe-Bf complex and EPR spectra was noted after sample was cooled to -125 °C.

To measure the gram susceptibility (χ_g) of the complex using Guoy's method, 70 mg of the complex was filled in the glass tube (Guoy's tube) and measurements were conducted using $[Ni(en)_3][S_2O_3]$ as a calibrant. First the instrument was set to zero by inserting a blank Guoy's tube and then 92.2 mg of the calibrant was filled in the tube and the reading of the instrument was noted. The value of 'χ_g' for $[Ni(en)_3][S_2O_3]$ was 0.00001103 emu/gm; using this value, 'C' was calculated employing the following equation:

$$\chi_g = CL\frac{R-R_0}{m \times 10^9} \qquad \text{(S4)}$$

In equation S4, 'C' is the calibration constant calculated using the calibrant whose χ_g is known, 'L' is the length of the sample in the Guoy's tube in cm (≥1.5 cm), 'R' is the balance reading for the calibrant and sample (after calculating C using calibrant), 'R_o' is the balance reading in the absence of sample (the blank), and 'm' is the sample mass in g.

After calculating the value of 'C', the 'χ_g' for the complex was calculated and from the value of χ_g, the molar susceptibility (χ_m) of the complex was obtained using the following equation:

$$\chi_m = \chi_g \times M \qquad \text{(S5)}$$

where 'M' is the molecular weight of the complex in g/mol. The molecular weight of the complex was deduced from the possible structures of the Fe-Bf complex based on stoichiometric studies (Figure 7 in the manuscript).

The 'χ_m' can be used to calculate the effective magnetic moment (μ_{eff}) using the following relation in Bohr Magnetons:

$$\mu_{eff} = 798\sqrt{\chi_m} \times T \qquad \text{(S6)}$$

where 'χ_m' is measured in m^3/mol and 'T' is measured in Kelvin.

III. UV-VIS SPECTROSCOPY STUDIES:

A. GENERAL PROCEDURE FOR THE STUDY OF INTERACTION BETWEEN METAL SALTS AND Bf:

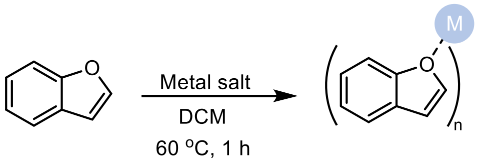

A 1-dram vial (VWR glass vials, 470151-622) equipped with a PTFE-coated stir bar (VWR spinbar micro, 3 x 10 mm, 58948-375) was charged with DCM (1.00 mL). To the reaction vial metal salt (0.050 mmol, 50.0 mM) and Bf (0.050 mmol, 50.0 mM) were added. The vial was fitted with a cap and the reaction was stirred at 300 rpm for 1 h at 60 °C. After stirring, 4 µL of the prepared solution was added to a quartz cuvette containing 1 mL of DCM. The UV-vis spectrum was collected from 250 nm through 800 nm for each of the prepared samples individually. Additionally, control samples for the iron salt and Bf were also recorded.

B. GENERAL PROCEDURE FOR THE STUDY OF THE STABILITY OF THE Fe-Bf COMPLEX UPON HEATING

Five 20 mL scintillation vials equipped with PTFE-coated stir bars were each charged with 10 mL of a 50 mM $FeCl_3$ solution in DCM. To the vials varying amount of Bf (50 mM, 100 mM, 150 mM, 250 mM and 500 mM) were added to obtain a mixture of $FeCl_3$ and Bf in ratios of 1:1, 1:2, 1:3, 1:5 and 1:10, respectively (Table S1). The vials were fitted with caps and the reactions were stirred at 300 rpm for 1 h at 60 °C. After stirring, 1.00 mL of the complex solution from each of the above solutions was transferred to three 1-dram vials (VWR glass vials, 470151-622) equipped with a PTFE-coated stir bar (VWR spinbar micro, 3 x 10 mm, 58948-375). Identical solutions were then stirred at 300 rpm for 1 h at different reaction temperatures (60 °C, 80 °C, and 100 °C) for 1 h. In order to achieve high temperature stirring of the complex in DCM at 80 and 100 °C, the vials were sealed using electrical tape and closed caps, rather than the septa-fitted caps. After stirring was completed, the vials were cooled down to room temperature. 10 µL of the prepared solution was added to a quartz cuvette containing 1.00 mL of DCM and the UV-vis absorption spectrum was collected from 250 nm through 800 nm for each of the prepared samples individually.

Table S1. Various proportions of $FeCl_3$ and Bf were employed to form the complex.

Entry	$FeCl_3$ (mM)	Benzofuran (mM)	Ratio
1	50	50	1:1
2	50	100	1:2
3	50	150	1:3
4	50	250	1:5
5	50	500	1:10

C. GENERAL PROCEDURE FOR THE STUDY OF THE STABILITY OF THE Fe-Bf COMPLEX UPON IRRADIATION:

Five 20 mL scintillation vials equipped with PTFE-coated stir bars were each charged with 10 mL of a 50 mM $FeCl_3$ solution in DCM. To the vials varying amount of Bf (50 mM, 100 mM, 150 mM, 250 mM and 500 mM) were added to obtain a mixture of $FeCl_3$ and Bf in ratios of 1:1, 1:2, 1:3, 1:5 and 1:10, respectively (Table S1). The vials were fitted with caps and the reactions were stirred at 300 rpm for 1 h at 60 °C. After stirring, 1.00 mL of the complex solution from each of the above solutions was transferred to three 1-dram vials (VWR glass vials, 470151-622) equipped with a PTFE-coated stir bar (VWR spinbar micro, 3 x 10 mm, 58948-375). Identical solutions were then stirred at 300 rpm for 1 h while being irradiated at different wavelengths (390 nm, 456 nm, and 525 nm). After stirring was completed, the vials were cooled to room temperature. 20 µL of the prepared solution was added to a quartz cuvette containing 1.00 mL of DCM and the UV-vis absorption data was collected from 250 nm through 800 nm for each of the prepared samples individually.

D. GENERAL PROCEDURE FOR THE STUDY OF THE STABILITY OF THE Fe-Bf COMPLEX IN DIFFERENT SOLVENTS:

In the experimental set-up 7.5 mM solution of $FeCl_3$ and a 7.5 mM solution of Bf were prepared in two different DCM: methanol solvent system (9:1 and 1:1; v/v ratio). Then, six 1-dram vials (VWR glass vials, 470151-622) equipped with a PTFE-coated stir bar (VWR spinbar micro, 3 x 10 mm, 58948-375) were charged with 2.00 mL of the 7.5 mM $FeCl_3$ solution. To the reaction vials some of the Bf stock solution was added in volumes to obtain different ratios of Fe to Bf as shown in Table S2. The vials were fitted with a cap and were stirred at 300 rpm for 1 h at 60 °C. After stirring was completed, the vials were cooled down to room temperature. 10 µL of the prepared solution was added to a quartz cuvette

containing 1.00 mL of DCM and the UV-vis spectrum was collected from 250 nm through 800 nm for each of the prepared samples individually. In addition, UV-vis spectrum was collected for $FeCl_3$ and Bf solutions as controls. Similarly, the experiment was repeated at higher concentration (50 mM) as shown in Table S2.

Table S2. Calculations for the preparations of the Fe-Bf complex for the stability studies.

Entry	7.5 mM $FeCl_3$ (mL)	Benzofuran (μl)	Ratio
1	2	1.6	1:1
2	2	3.2	1:2
3	2	4.8	1:3
4	2	6.4	1:4
5	2	8	1:5
6	2	16	1:10

Entry	50 mM $FeCl_3$ (mL)	Benzofuran (μl)	Ratio
1	2	11	1:1
2	2	22	1:2
3	2	33	1:3
4	2	44	1:4
5	2	55	1:5
6	2	110	1:10

In a different experiment, the stability of the Fe-Bf complex was evaluated in the presence of various organic solvents. The complex was prepared using 50 mM $FeCl_3$ and 50 mM Bf in DCM after stirring at 300 rpm for 1 h at 60 °C (Section III-A). After stirring was completed, the vials were cooled down to room temperature. For UV-vis analysis of the different samples, 20 µL of the complex was added to a quartz cuvette charged with DCM: solvent (1:1; v/v ratio). The sample was analyzed in the presence of different organic solvents including cyclohexane, methanol, toluene, DCM, ethyl acetate, dimethylformamide, acetonitrile, dimethyl sulfoxide, tetrahydrofuran, *N*, *N*-dimethylacetamide and diethyl ether. The UV-vis spectrum was collected from 250 nm through 800 nm for each of the prepared samples.

E. GENERAL PROCEDURE FOR THE STUDY OF THE FORMATION OF THE Fe-Bf COMPLEX AT DIFFERENT STOICHIOMETRIC RATIOS OF Fe AND Bf

To a 20 mL scintillation vial, 50 mM stock solution of $FeCl_3$ and 50 mM stock solution of Bf were prepared in DCM. The stock solutions were then mixed in the correct proportions to obtain Fe:Bf ratios of 1:1, 1:2, 1:3, 1:4, 1:5 and 1:10. The vials were then stirred at 300 rpm for 1 h at 60 °C. After stirring was completed, the vials were cooled down to room temperature.10 µL of the prepared solution was added to a quartz cuvette containing 1.00 mL of DCM and the UV-vis absorption spectrum was collected from 250 nm through 800 nm for each of the prepared samples individually.

F. GENERAL PROCEDURE FOR THE MOLAR RATIO METHOD AND JOB PLOT

The stoichiometry of the Fe-Bf complex (50 mM) was investigated using two different UV-vis absorption spectroscopy methods. Two different plots were drawn including a Molar Ratio Plot and a Job Plot.

In the molar ratio method, the absorption of the complex is plotted against the molar ratio of the two interacting species while keeping the concentration of Fe constant. A 50 mM stock solution of $FeCl_3$ was prepared in DCM. 1-dram vials (VWR glass vials, 470151-622) equipped with a PTFE-coated stir bar (VWR spinbar micro, 3 x 10 mm, 58948-375) were charged with 1.0 mL of the $FeCl_3$ solution. To prepare the Fe-Bf complex with varying Fe to Bf ratios (1:1, 1:2, 1:3, 1:4, 1:5, 1:6, 1:7, 1:8, 1:9, and 1:10), the vials were subjected to increasing concentrations of Bf ranging from 50 mM, 100 mM, 150 mM, 200 mM, 250 mM, 300 mM, 350 mM, 400 mM, 450 mM and 500 mM respectively. The vials were then stirred at 300 rpm for 1 h at 60 °C. After stirring was completed, the vials were cooled down to room temperature. 10 µL of the prepared solution was added to a quartz cuvette containing 1.0 mL of DCM and the UV-vis data was collected from 250 nm through 800 nm for each of the prepared samples individually. The variation in the absorbance intensity of the absorption peak at 554 nm were observed and plotted with respect to the molar ratio of the complex and the stoichiometry of the complex formed was deduced from the position of breaks in the absorption curve.

In the Job plot method, the Fe-Bf complex was prepared using different mole fractions of the $FeCl_3$ and Bf while keeping the molar concentration of the complex constant. A 100 mM stock solution of $FeCl_3$ and a 100 mM stock solution of Bf were prepared in DCM. Next, 1-dram vials (VWR glass vials, 470151-622) equipped with a PTFE-coated stir bar (VWR spinbar micro, 3 x 10 mm, 58948-375) were charged with $FeCl_3$ and Bf in proportions given in Table S3. The vials were then stirred at 300 rpm for 1 h at 60 °C. After stirring was completed, the vials were cooled down to room temperature. 10 µL of the prepared solution was added to a quartz cuvette containing 1.0 mL of DCM and the UV-vis data was collected from 250 nm through 800 nm for each of the prepared samples individually. Variations in the absorbance intensity of the absorption peak at 554 nm were observed. The Job plot was deduced from the absorption studies by assigning the x-axis of the plot to the mole fraction of Fe(III), and the y-axis to the absorbance intensity of the absorption maxima at 554 nm. The stoichiometry of the complex was deduced from the maxima of the Job plot curve.

Table S3. Various proportions of $FeCl_3$ and Bf used to form the samples for the Job plot.

Entry	$FeCl_3$ (mM)	Benzofuran (mM)
1	0	100
2	10	90
3	15	85
4	20	80
5	25	75
6	30	70
7	35	65
8	40	60
9	50	50
10	60	40
11	70	30
12	80	20
13	90	10
14	100	0

G. GENERAL PROCEDURE FOR THE EVALUATION OF THE BINDING CONSTANT FOR THE COMPLEX USING THE BENESI-HILDEBRAND RELATION

Furthermore, the extent of the binding of Bf toward Fe(III) ion is calculated from an experimental plot of the absorption data using the Benesi–Hildebrand relation. A vial was charged with 2.0 mL of 150 mM Bf solubilized in DCM and 10 µL of this was added to a quartz cuvette charged with 1.0 mL DCM and the UV-vis spectrum was noted. To the vial, aliquots of $FeCl_3$ (0-50 mM) were added and 10 µL of the formed solution was drawn after each addition into the cuvette charged with 1.0 mL DCM and the variations in absorption spectrum were noted. The variation in the absorption band at 554 nm was observed and a linear fitted curve was then obtained with logarithm of concentration of $FeCl_3$ on the x-axis and logarithm of the variation in the absorption intensity plotted on the y-axis. The binding constant (K_b) was determined from the intercept value of the linear fitting absorption curve using the following Benesi-Hildebrand equation:

$$log\frac{(A- A_o)}{(A_f- A_o)} = \log[Fe] + \log K_b \qquad \text{(S7)}$$

in which A_0, A and A_f are the absorption values, in the absence of, at the intermediate, and at the saturation of the interaction of ferric ion, respectively. For the experiment, UV-vis absorption spectrum of Bf was collected in the presence of increasing amounts of $FeCl_3$ until saturation was observed (Figure S6). The higher the value of the binding constant, the stronger the interaction between the metal ion and the ligand. Also, the experiment was conducted three times to validate the binding affinity of Bf towards ferric ions.

H. GENERAL PROCEDURE FOR THE STUDY OF THE SELECTIVITY OF $FeCl_3$ IN THE PRESENCE OF OTHER VOCS

The interfering VOCs chosen for the selectivity study were anthracene, 9-hydroxy fluorine, quinoline, triethylene glycol, catechol, salicylic acid, benzothiophene, dibenzofuran, 2, 3-dihydrobenzofuran, toluene, fluoranthene and 1-methyl naphthalene (Figure S1).

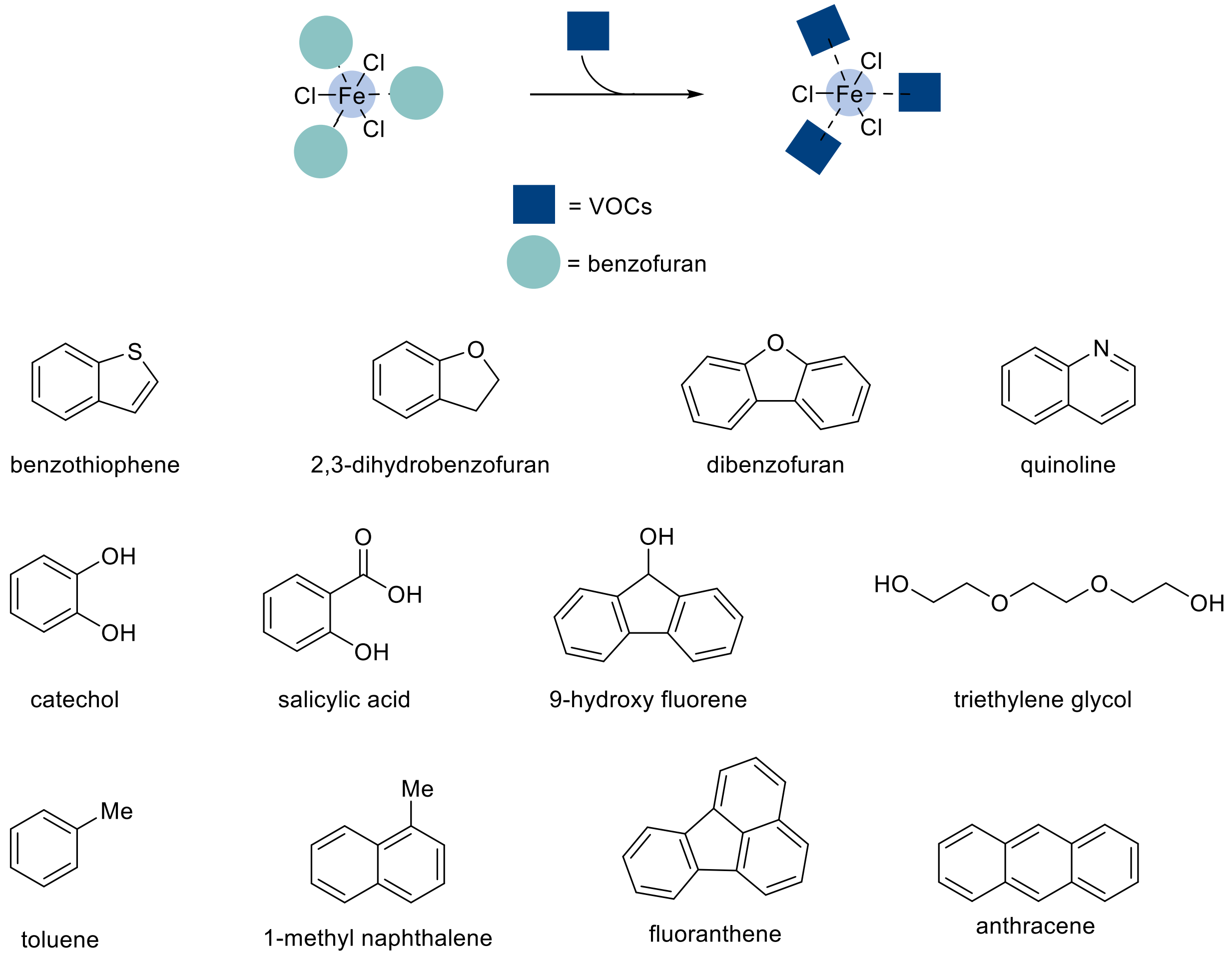

Figure S1: The binding activity of the potentially interfering VOCs with the Fe-Bf complex and chemical structures of all the VOCs investigated for their potential to displace Bf from Fe-Bf complex.

In this experiment $FeCl_3$ (0.1 mmol, 1.0 equiv.), Bf (0.3 mmol, 3.0 equiv.), and other VOC (0.3 mmol, 3.0 equiv.) were mixed in 2.0 mL DCM and were stirred at 300 rpm for 1 h at 60 °C. After stirring was completed, the vials were cooled down to room temperature. 10 µL of the prepared solution was added to a quartz cuvette containing 1.0 mL of DCM and UV-vis spectrum was collected from 250 nm through 800 nm for each of the prepared samples individually (Figure S7-S8). In addition, the controls were run along with the complex to gain insight into the possible interactions of the VOCs with the complex or $FeCl_3$. UV-vis spectra of the VOCs and $FeCl_3$ in the presence of other VOCs were recorded to analyze possible interactions of the VOC with $FeCl_3$. It was observed that some VOCs interacted with $FeCl_3$ evidenced from new peaks in the UV-vis absorption spectrum. In order to further investigate the interaction between the complex and those VOCs (quinoline and triethylene glycol), titration studies were conducted. Small aliquots (5 mM each) of the VOCs were added into the pre-formed complex solution (50 mM) stepwise and the absorption spectra were noted after each addition.

I. GENERAL PROCEDURE FOR THE DIRECT APPLICATION OF Fe-INCORPORATED BIOCHAR FOR Bf INHIBITION:

In this experiment, 100 mg of the biochar was solubilized in 10 mM $FeCl_3$ solution prepared in DCM. The solution was then stirred vigorously at 1700 rpm for 24 h for adsorption to take place. Meanwhile, known concentrations of $FeCl_3$ were spectroscopically analyzed to obtain a calibration plot as shown in Figure S15. The solution was then filtered using a Buchner funnel with a fritted disc (VWR, 10545-880) and the concentration of the Fe(III) ions in the filtrate was calculated using the calibration plot. The adsorption capacity of the biochar was then calculated by subtracting the amount in the filtrate from the initially fed concentration. The Fe-treated biochar was analyzed using TGA and FTIR after drying the residue in oven at 80 °C for 15 minutes. The finely grounded dry material (10-30 mg) was weighed into 70 µl Al_2O_3 pan with an Al lid. The samples were then heated under a flow of 20 mL/min N_2 from 25 °C to 1000 °C at a ramp rate of 10 °C/min. The residue was also analyzed using FTIR spectroscopy from 400-4000 cm^{-1}. Biochar and $FeCl_3$ were also analyzed individually by TGA and FTIR as controls. The residue was then solubilized in DCM followed by addition of Bf to form Fe-Bf complex. From the calibration plot, the adsorption capacity was obtained to be 13.02 mg/g of biochar which means that out of the 10 mM $FeCl_3$ which was subjected to 100 mg of biochar, 4 mM of the $FeCl_3$ was adsorbed.

Therefore, 12 mM (3 equivalents with respect to adsorbed $FeCl_3$) Bf was added and the solution was stirred for 1 h at 60 °C. Stirring yielded a purple-colored complex which was spectroscopically analyzed by observing the UV-vis absorption spectra from 250 nm through 800 nm.

II. TGA-FTIR STUDIES:

A. PROCEDURE TO CONFIRM BINDING OF Bf TO $FeCl_3$

A sample of $FeCl_3$ and Bf was prepared by dissolving $FeCl_3$ (16.2 mg, 0.100 mmol) and Bf (10.8 µL, 0.100 mmol) in DCM (3.00 mL). The solution was stirred at room temperature for 1 h before being concentrated *in vacuo* resulting in a dark purple solid. A background spectrum for the IR was taken and the TGA pan was tared prior to loading the sample in the furnace. A portion (16.0 mg) of the sample was transferred to a high-temperature Pt TGA pan. The pan was then loaded into the furnace and the FTIR series data was collected while the sample was heated using the following experimental setup:

- Ramp rate of 20 °C/min to 40 °C
- Isothermal hold at 40 °C for 10 minutes (to ensure that all DCM evaporated)
- Ramp rate of 20 °C/min to 1000 °C

In addition, a control sample of Bf (10.8 µL) in 200 µL DCM was also analyzed using the above mentioned TGA-FTIR method.

B. PROCEDURE FOR THERMAL ANALYSIS OF THE DEGRADATION OF THE Fe-Bf COMPLEX

The sample was prepared as in the TGA-FTIR procedure described above, except that the experiment setup for heating the sample was adjusted to the following method:

- Ramp rate of 20 °C/min to 40 °C
- Isothermal hold at 40 °C for 10 minutes (to ensure that all DCM evaporated)
- Ramp rate of 20 °C/min to 200 °C
- Isothermal hold at 200 °C for 40 minutes (to assess stability at 200 °C)
- Ramp rate of 20 °C/min to 1000 °C

III. SPECTROSCOPIC DATA

A. STABILITY OF THE Fe-Bf COMPLEX AT DIFFERENT TEMPERATURES AND WAVELENGTHS OF IRRADIATION

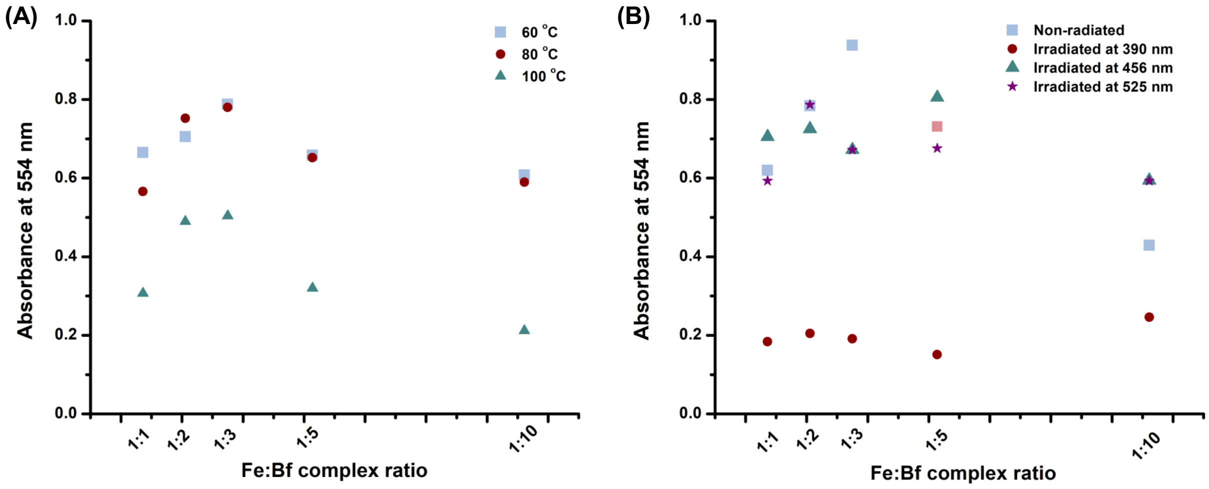

Figure S2. Plot of the intensity of absorbance at 554 nm (characteristic of complex formation) for the 50 mM Fe-Bf complex in different ratios of Fe:Bf subjected to varying (A) temperatures (60 °C, 80 °C, and 100 °C) and (B) irradiation conditions (390 nm, 456 nm and 525 nm).

B. STABILITY OF COMPLEX IN DCM/METHANOL SYSTEM ESTABLISHED USING UV-Vis SPECTROSCOPY

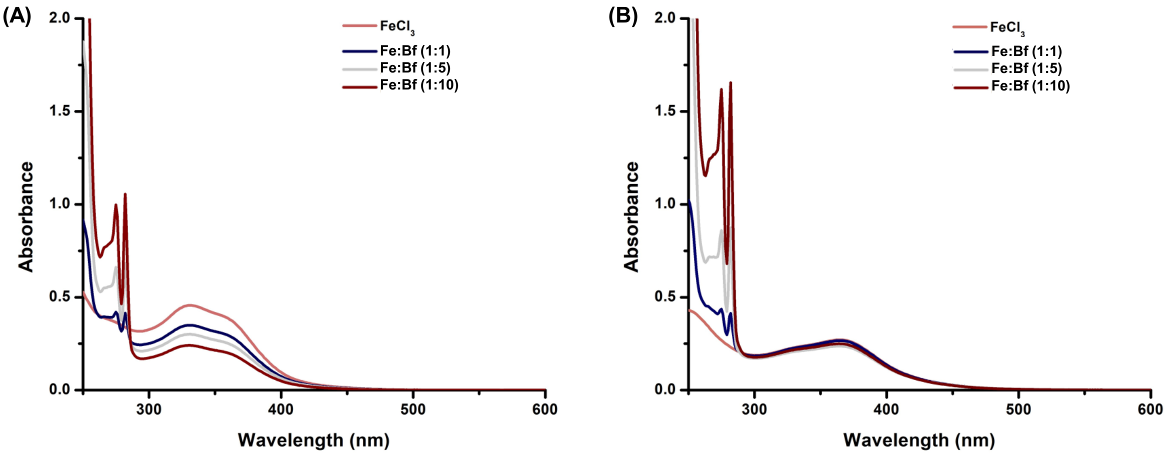

Figure S3. UV-vis absorption spectrum of Fe-Bf complex (50 mM) in different ratios in (A) DCM:methanol (9:1 v/v) and (B) DCM:methanol (1:1 v/v) solvent system.

As observed in Figure S3, the peak at 550-550 nm was not detected in the presence of methanol. This suggests that no Fe-Bf complex was formed or that the Fe-Bf complex got destabilized.

Subsequently, the stability of Fe-Bf in the presence of different homogeneous systems was established. While the vast majority of these experiments were conducted in DCM, a variety of solvents such as acetonitrile (MeCN), dimethylformamide (DMF), and dimethylsulfoxide (DMSO) can act as Lewis basic ligands just like Bf; therefore, these solvents as well as a range of protic and commonly used aromatic solvents were evaluated for competitive binding to Fe using UV-vis spectroscopy (Figure S4). The Fe-Bf complex was pre-made in DCM and subsequently exposed to a variety of common organic solvents in a 1:1 v/v ratio (Figure S4). While the Fe-Bf complex was stable in the presence of cyclohexane, toluene, and ethyl acetate, it was unstable in all other examined solvents, including DMF, acetonitrile, DMSO, tetrahydrofuran, dimethylacetamide, and diethylether.

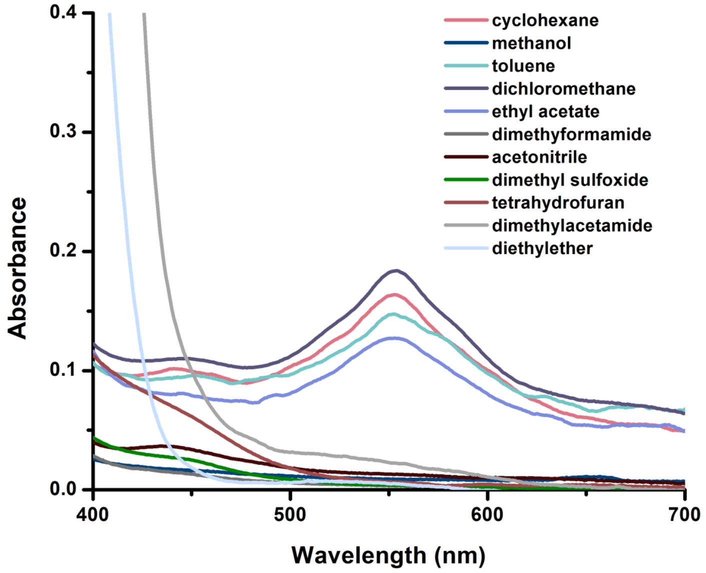

Figure S4. UV-visible absorption spectra of pre-formed Fe-Bf complex in the presence of various solvent systems. The pre-formed Fe-Bf complex (50 mM) was diluted 250 times in 1 mL DCM:Solvent (1:1, v/v) system prior to UV-vis analysis. In the above scheme 'm' is the number of Bf units and 'n' is the number of chlorine atoms.

Further studies of the $FeCl_3$ and Bf system focused on investigating the effect of varying their relative concentrations on coordination (Figure S5). Increasing the concentration of Bf in the system relative to $FeCl_3$ appeared to increase the intensity of absorbance at 554 nm, suggesting a higher concentration of the Fe-Bf complex was formed.

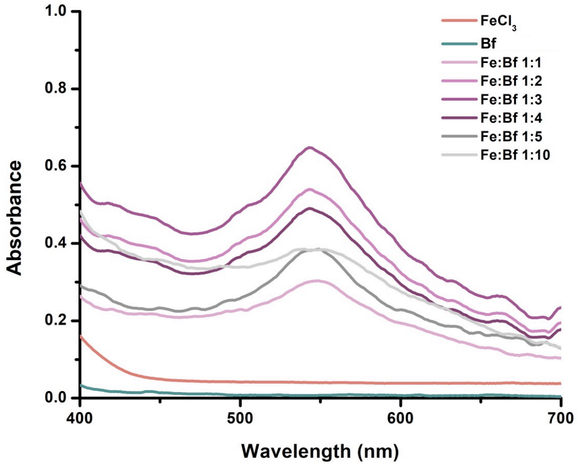

Figure S5. UV-vis absorption spectra of 50 mM $FeCl_3$ (black), Bf (very light blue), and mixtures of $FeCl_3$ and Bf at 1:1 (dark blue), 1:2 (light blue), 1:3 (brown), 1:4 (gray), 1:5 (pink), and 1:10 (light green) ratios stirred for 1 h in DCM at 60 °C.

C. BENESI-HILDEBRAND PLOT

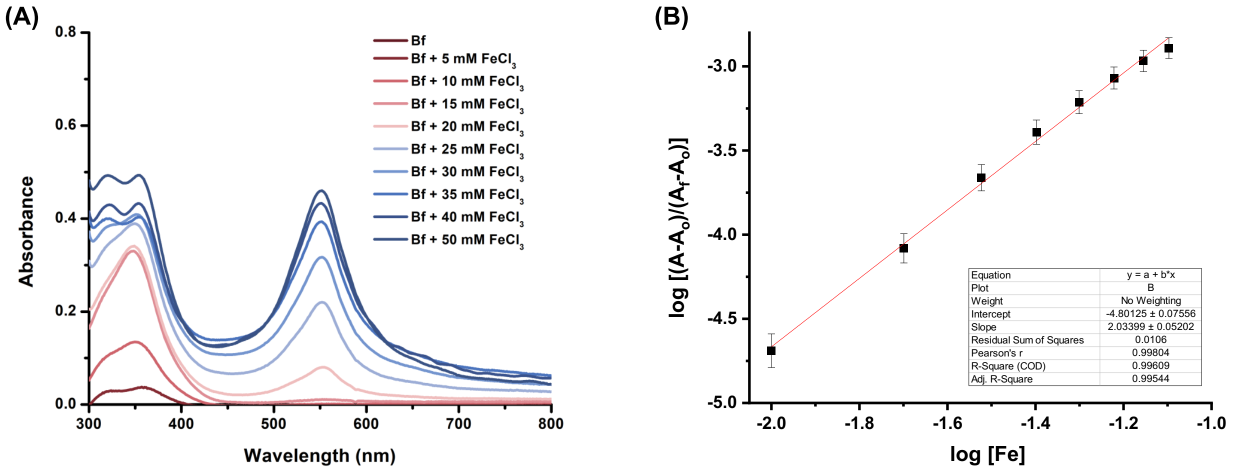

Figure S6. (A) UV-visible absorption spectrum of Bf in the presence of increasing concentrations of $FeCl_3$, (B) Benesi-Hildebrand plot for titration studies.

D. SELECTIVITY OF $FeCl_3$ IN BINDING DIFFERENT VOCs AND THE Fe-Bf COMPLEX UPON ADDITION OF DIFFERENT VOCs

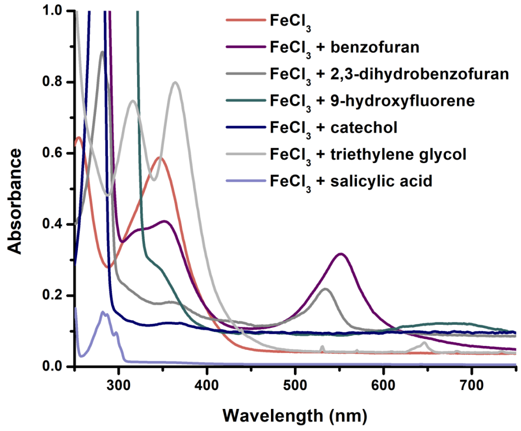

Figure S7. UV-vis absorption spectra of $FeCl_3$ in the presence of different O-containing VOCs: Bf, 2,3-dihydrobenzofuran, 9-hydroxy fluorene, catechol, triethylene glycol and salicylic acid.

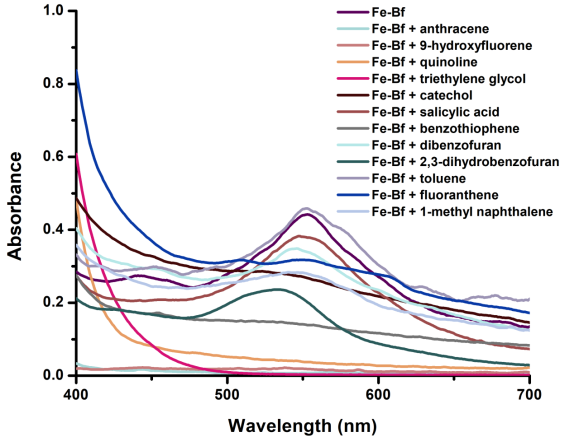

Figure S8. UV-visible absorption spectra of Fe-Bf complex in the presence of 3 equiv. of various VOCs.

Table S4. Summary of the Selectivity Data for the Complex in Presence of Various VOCs Obtained Using UV-vis Absorption Spectroscopy.

Entry	No new peak	New peak(s) formed	
1	anthracene[a]	quinoline[a,c]	(365 nm)
2	9-hydroxy fluorene[a]	triethylene glycol[a,c]	(316 and 365 nm)
3	catechol[a]	2,3-dihydrobenzofuran[a,c]	(535 nm)
4	salicylic acid[b]	toluene[b]	(458 nm)
5	benzothiophene[a]		
6	dibenzofuran[b]		
7	fluoranthene[b]		
8	1-methyl naphthalene[b]		

[a] Complex is not stable in the presence of these VOCs.

[b] Complex is stable in the presence of these VOCs.

[c] Replaced benzofuran from the complex and formed new complex.

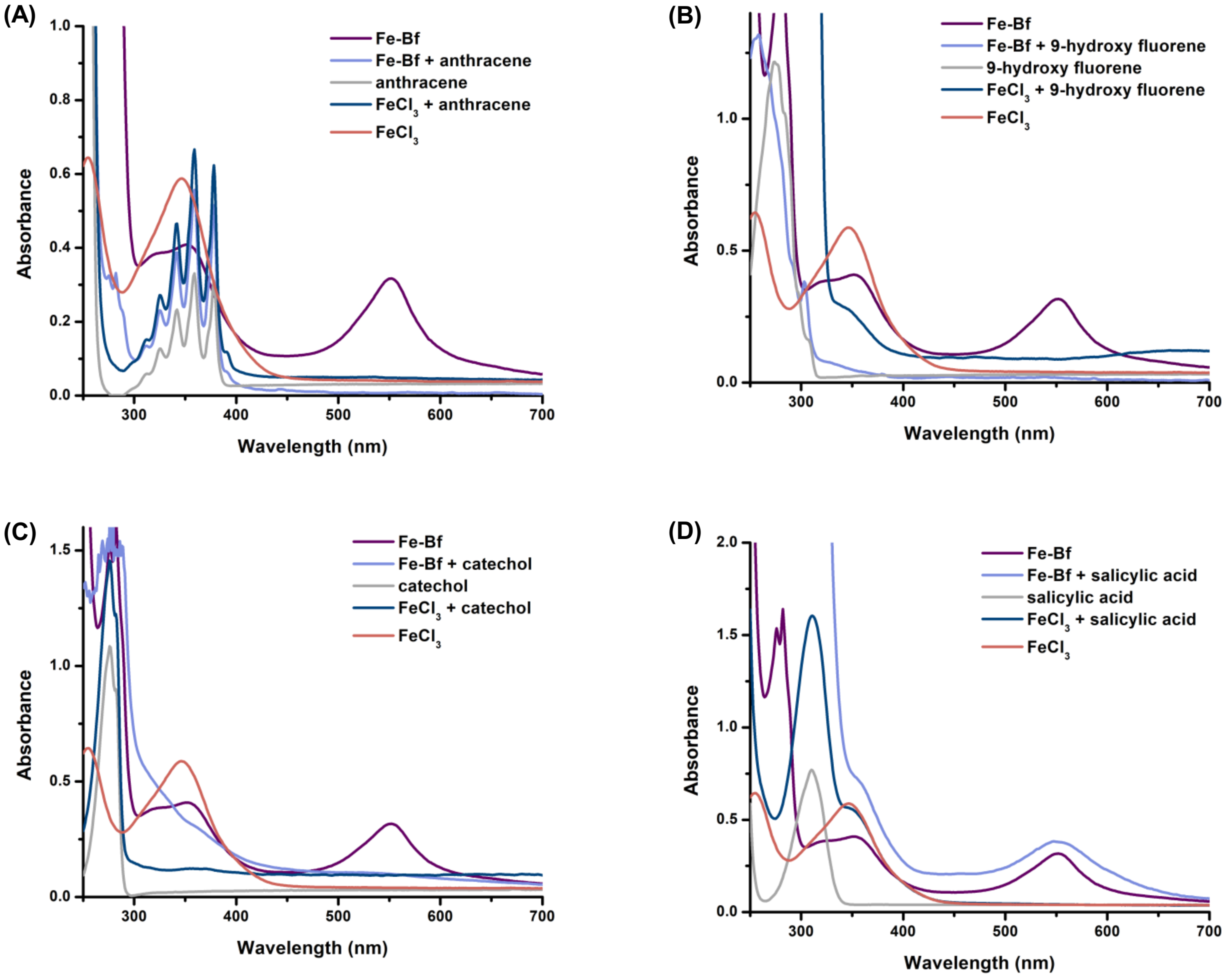

Figure S9. UV-vis absorption spectra of Fe-Bf complex in the presence of (A) anthracene, (B) 9-hydroxyfluorene, (C) catechol, and (D) salicylic acid along with controls of VOCs, VOCs + $FeCl_3$ and $FeCl_3$ solubilized in DCM displaying distinct absorption bands along with disappearance of complex absorption peak at 554 nm except for salicylic acid.

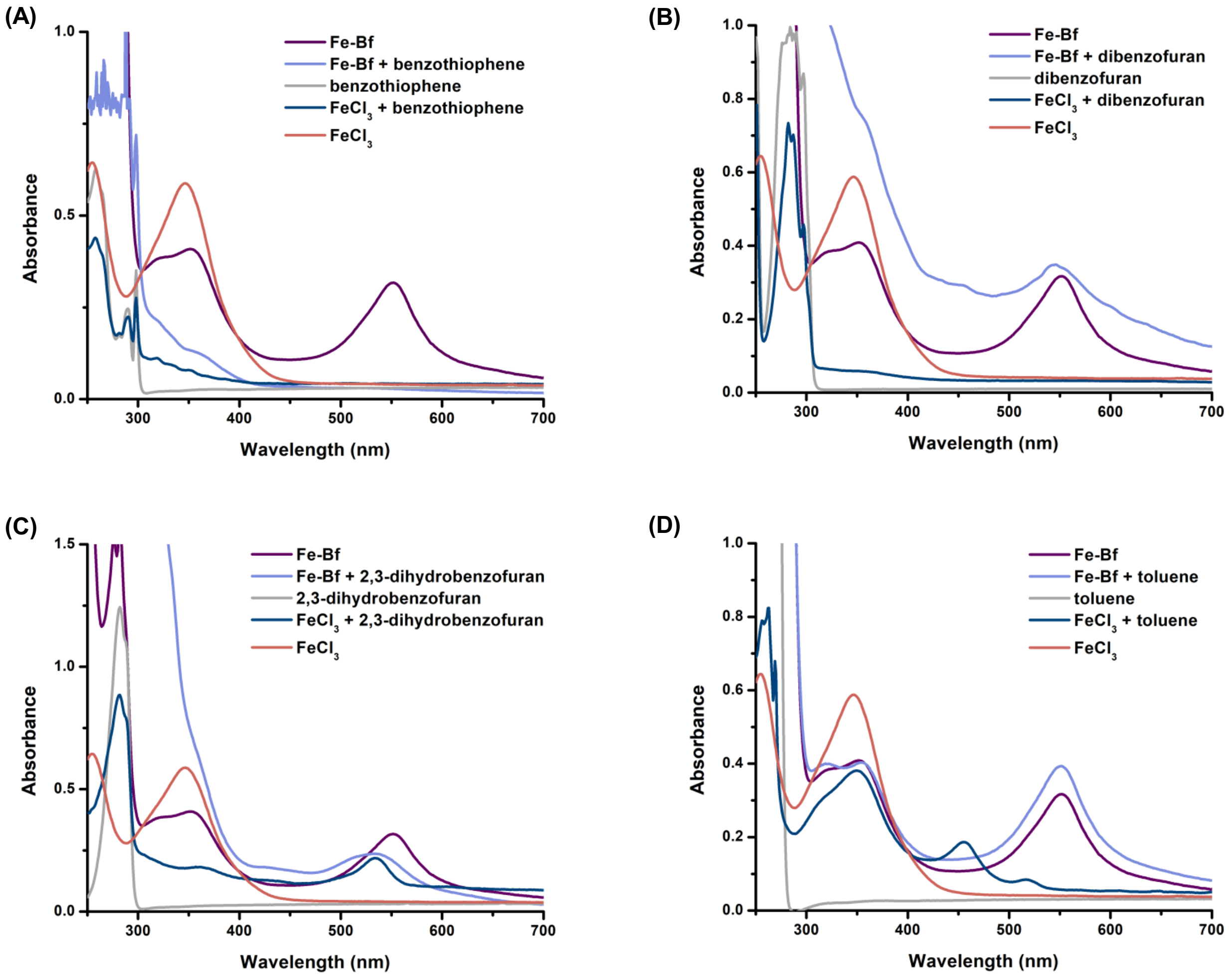

Figure S10. UV-vis absorption spectra of Fe-Bf complex in the presence of (A) benzothiophene, (B) dibenzofuran, (C) 2,3-dihydrobenzofuran, and (D) toluene along with controls of VOCs, VOCs + $FeCl_3$, and $FeCl_3$ solubilized in DCM displaying disappearance of the complex absorption peak at 554 nm in the presence of benzothiophene.

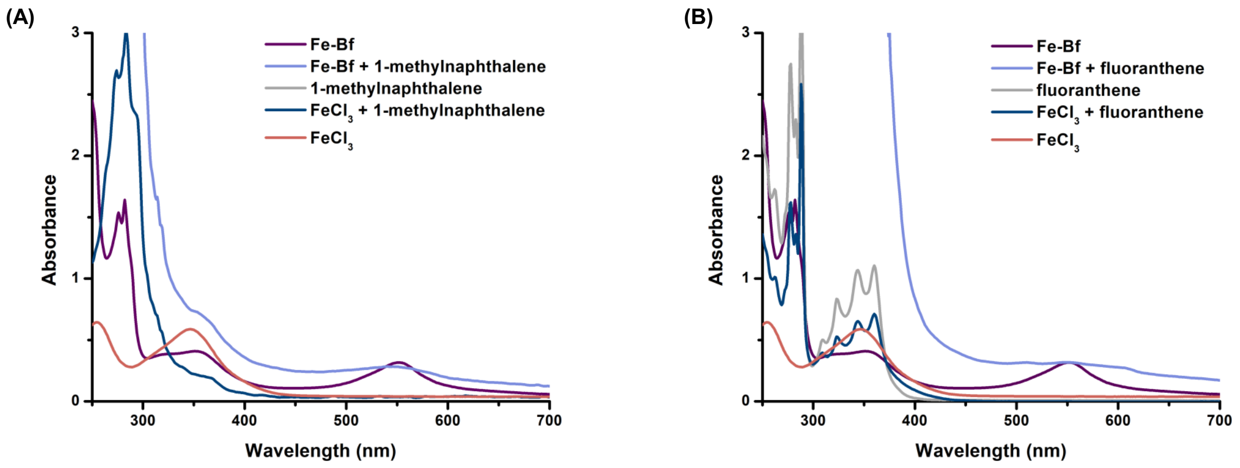

Figure S11. UV-vis absorption spectra of Fe-Bf complex in the presence of (A) 1-methyl naphthalene, and (B) fluoranthene along with controls of VOCs, VOCs + $FeCl_3$ and $FeCl_3$ solubilized in DCM.

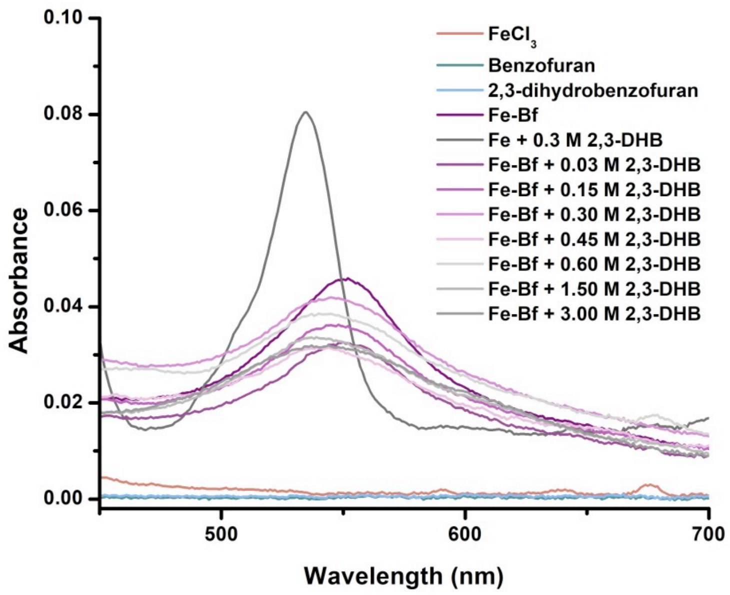

Figure S12. UV-vis absorption spectra of the Fe-Bf complex in the presence of increasing concentrations of 2,3-dihydrobenzofuran.

E. DIRECT APPLICATION OF Fe-INCORPORATED BIOCHAR FOR Bf INHIBITION:

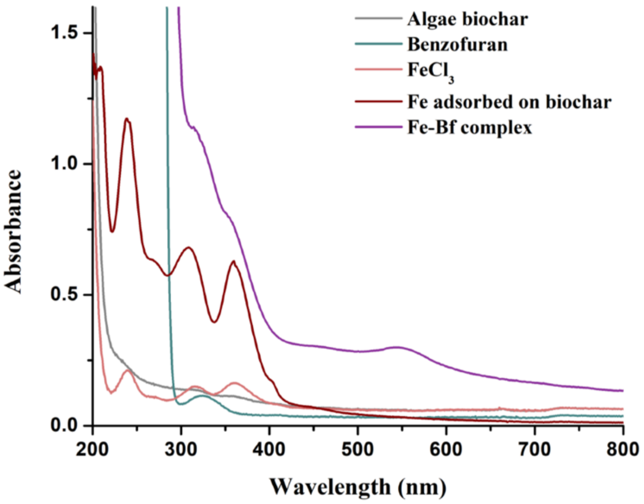

Figure S13. UV-vis absorption spectra of Algae biochar (gray), Bf (green), $FeCl_3$ (pink), Fe-rich biochar (maroon), and Fe-Bf complex (purple) formed on the surface of biochar showing distinct absorption bands.

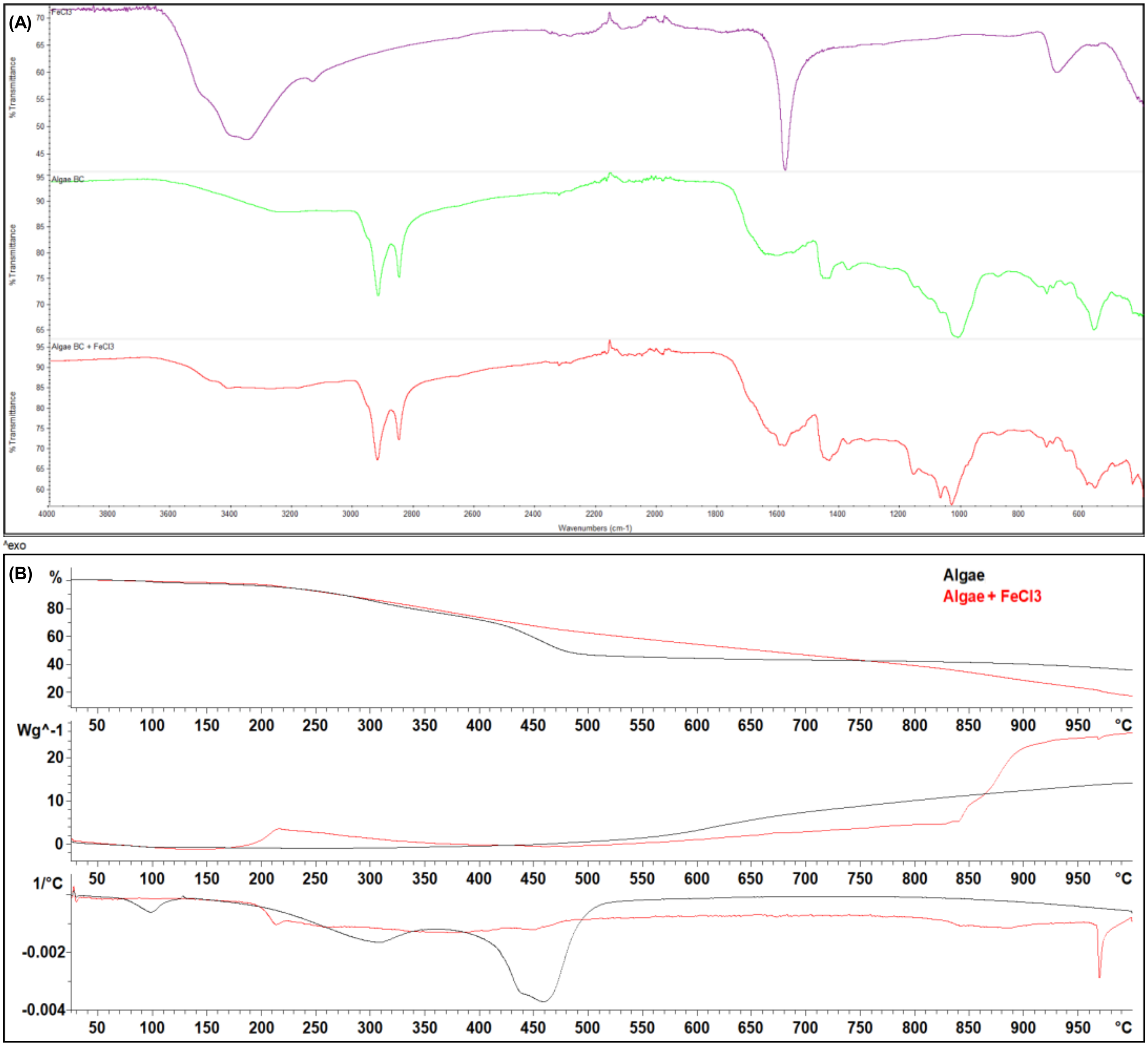

Figure S14: (A) FTIR spectra of $FeCl_3$, Algae biochar and Algae biochar + $FeCl_3$ displaying distinct stretching and vibration frequencies. **(B)** TGA-DSC and DTG comparison graphs for Algae biochar in the absence and presence of $FeCl_3$ displaying unique thermal degradation pathway in the presence of $FeCl_3$ suggesting physical adsorption of Fe species on the surface of biochar.

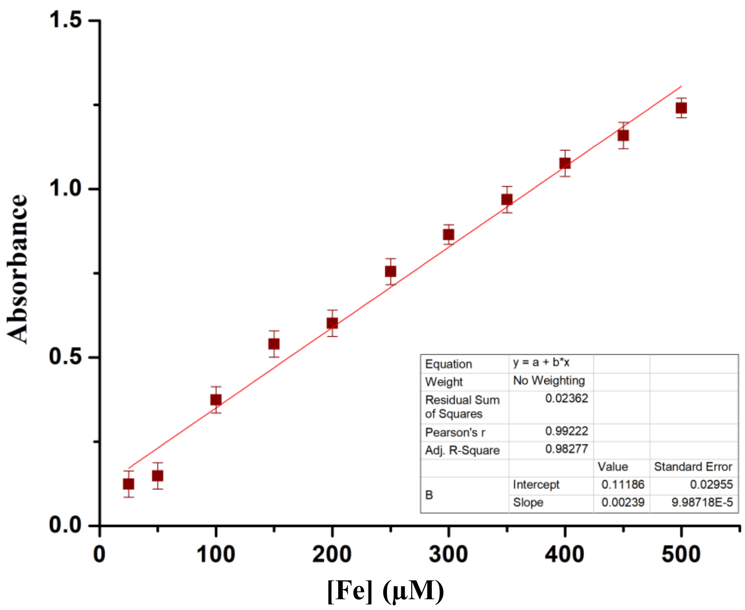

Figure S15. Calibration plot of $FeCl_3$ displaying linear progression coefficient of 0.99557 with increasing concentration of Fe(III) ions.

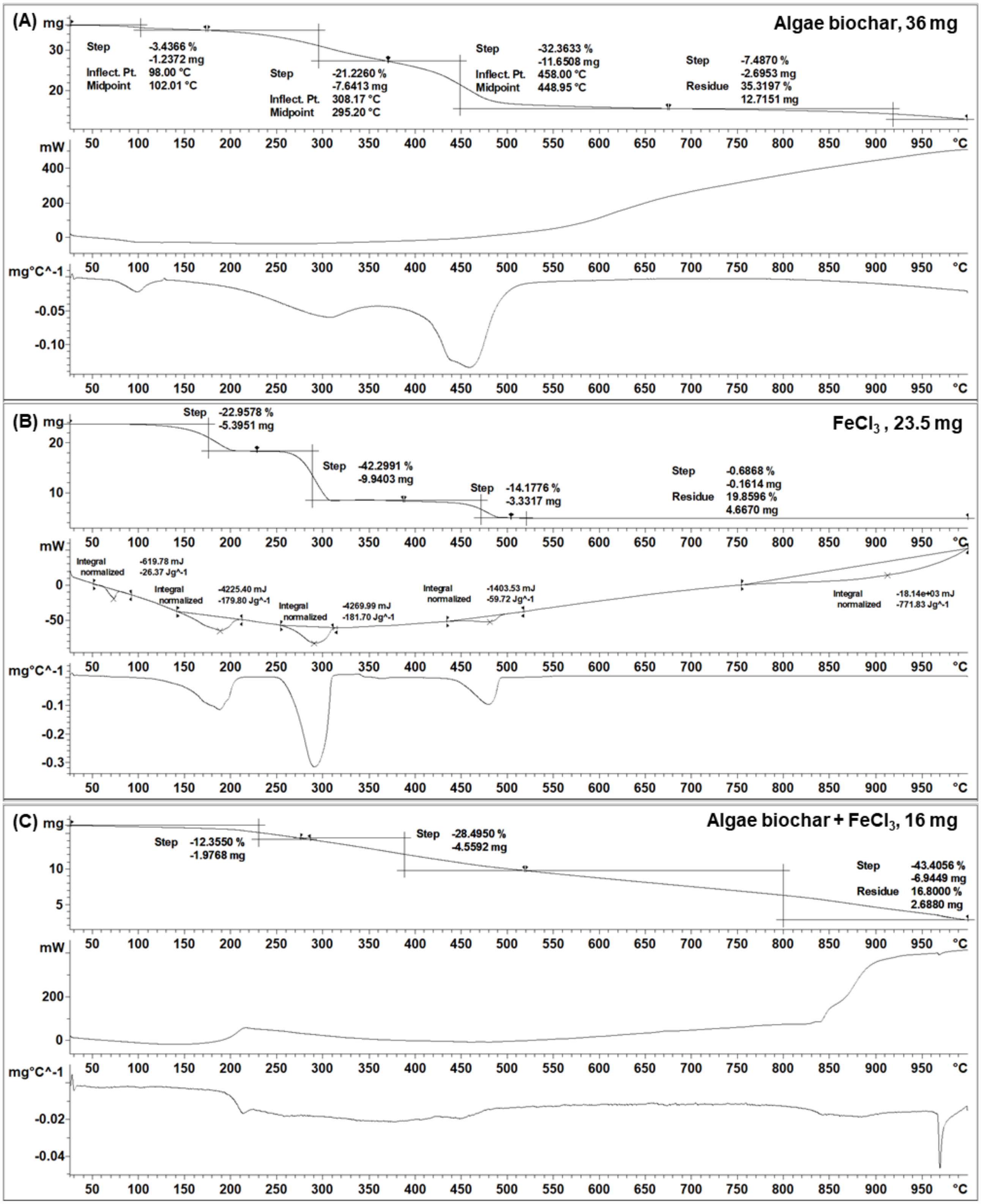

Figure S16: TGA, DSC and DTG curves of **(A)** Algae biochar, **(B)** $FeCl_3$, and **(C)** Algae biochar in the presence of $FeCl_3$ displaying distinct thermal degradation pathways as well as characteristic heat flow signatures when heated up to 1000 °C at a ramp rate of 10 °C/min.

REFERENCES

1. Delley, B. From Molecules to Solids with the DMol3 Approach. *J. Chem. Phys.* **2000**, *113*, 7756-7764. https://doi.org/10.1063/1.1316015.
2. Perdew, J. P.; Burke, K.; Ernzerhof, M. Generalized Gradient Approximation Made Simple. *Phys Rev Lett* **1996**, *77*, 3865-3868. http://dx.doi.org/10.1103/PhysRevLett.77.3865
3. Grimme, S. Density functional theory with London dispersion correction. *Wiley Interdiscip. Rev.: Comput. Mol. Sci.* **2011**, *1*, 211-228. https://doi.org/10.1002/wcms.30.
4. Klamt, A.; Schüürmann, G. COSMO: A new approach to dielectric screening in solvents with explicit expressions for the screening energy and its gradient. *J. Chem. Soc., Perkin Trans. 2.* **1993**, 799-805. http://dx.doi.org/10.1039/P29930000799.
5. Andzelm, J.; Kölmel, C.; Klamt, A. Incorporation of solvent effects into density functional calculations of molecular energies and geometries. *J. Chem. Phys.* **1995**, *103,* 9312-9320. https://aip.scitation.org/doi/abs/10.1063/1.469990.
6. Goldfarb, D.; Stoll, S. (Eds). *EPR spectroscopy: Fundamentals and methods*; John Wiley & Sons Ltd: Chichester, **2018**.
7. Stoll, S. CW-EPR spectral simulations: Solid state. *Methods Enzymol*. **2015**, *563*, 121−142. https://doi.org/10.1016/bs.mie.2015.06.003.
